# The Histone Demethylase Jhdm1a Regulates Hepatic Gluconeogenesis

**DOI:** 10.1371/journal.pgen.1002761

**Published:** 2012-06-14

**Authors:** Dongning Pan, Chunxiao Mao, Tie Zou, Annie Y. Yao, Marcus P. Cooper, Victor Boyartchuk, Yong-Xu Wang

**Affiliations:** 1 Program in Gene Function and Expression and Program in Molecular Medicine, University of Massachusetts Medical School, Worcester, Massachusetts, United States of America; 2 Program in Gene Function and Expression and Department of Molecular Genetics and Microbiology, University of Massachusetts Medical School, Worcester, Massachusetts, United States of America; 3 Division of Cardiovascular Medicine, University of Massachusetts Medical School, Worcester, Massachusetts, United States of America; Karolinska Institutet, Sweden

## Abstract

Hepatic gluconeogenesis is required for maintaining blood glucose homeostasis; yet, in diabetes mellitus, this process is unrestrained and is a major contributor to fasting hyperglycemia. To date, the impacts of chromatin modifying enzymes and chromatin landscape on gluconeogenesis are poorly understood. Through catalyzing the removal of methyl groups from specific lysine residues in the histone tail, histone demethylases modulate chromatin structure and, hence, gene expression. Here we perform an RNA interference screen against the known histone demethylases and identify a histone H3 lysine 36 (H3K36) demethylase, Jhdm1a, as a key negative regulator of gluconeogenic gene expression. In vivo, silencing of Jhdm1a promotes liver glucose synthesis, while its exogenous expression reduces blood glucose level. Importantly, the regulation of gluconeogenesis by Jhdm1a requires its demethylation activity. Mechanistically, we find that Jhdm1a regulates the expression of a major gluconeogenic regulator, C/EBPα. This is achieved, at least in part, by its USF1-dependent association with the C/EBPα promoter and its subsequent demethylation of dimethylated H3K36 on the C/EBPα locus. Our work provides compelling evidence that links histone demethylation to transcriptional regulation of gluconeogenesis and has important implications for the treatment of diabetes.

## Author Summary

Histones are small proteins that are essential for packaging and ordering genetic information (DNA) into high-order chromatin structures. Methylation of specific lysine residues of histones alters chromatin structure, serving as an important epigenetic mechanism for regulation of gene expression. The dynamic nature of histone methylation is controlled by a balance of methyltransferases and demethylases. We have discovered here that the demethylase Jhdm1a negatively regulates gluconeogenesis (*de novo* glucose synthesis) through suppressing the expression of two rate-limiting gluconeogenic enzymes. Gluconeogenesis is required for maintaining blood glucose homeostasis; yet, in diabetes mellitus, this process is unrestrained and is a major contributor to hyperglycemia. Indeed, we have found that manipulation of Jhdm1a level in liver affects glucose production in normal mice and hyperglycemia in diabetic mice. Mechanistically, Jhdm1a actively removes dimethyl groups from histone H3K36 along the locus of a key gluconeogenic regulator, C/EBPα, which in turn results in decreased C/EBPα expression. Our findings thus identify histone demethylation as a novel regulatory mechanism for gluconeogenesis and have important implications for the treatment of diabetes.

## Introduction

Hepatic glucose production is critical for the maintenance of normal blood levels to meet whole-body fuel requirements. In the early phase of postabsorptive state, circulating glucose is supplied from breakdown of liver glycogen stores. When fasting progresses, gluconeogenesis, which utilizes non-carbohydrate precursors to *de novo* synthesize glucose, becomes the major form of hepatic glucose production [1], [2]. In both type 1 and type 2 diabetes, gluconeogenesis is exaggerated and contributes to hyperglycemia [3]–[5].

The rate of gluconeogenesis is largely determined by three rate-limiting enzymes, Phosphoenolpyruvate carboxykinase (PEPCK), fructose-1,6-bisphosphatase (FBP-1) and glucose 6-phosphatase (G6Pase). The levels of these gluconeogenic enzymes are controlled by hormonal signals, notably glucagon and glucocorticoids, and the opposing hormone insulin, at the transcription level. Key DNA elements responsible for the hormonal regulation have been well characterized on the promoters of PEPCK and G6Pase gene [6]–[9]. These elements serve as platforms for setting up a complex transcriptional machinery that includes transcription factors (e.g., CREB, FOXO1, FOXA2, C/EBPs, HNF4α, GR, Nur77) and co-factors (e.g., PGC-1α, CRTC2, SIRT1, p300/CBP, SRC-1), thereby driving gluconeogenic gene expression [10], [11]. Despite these tremendous progresses, the regulatory mechanisms upstream of this transcriptional network are incompletely understood. Furthermore, it is unclear how the chromatin landscape affects gluconeogenesis, what chromatin modifying enzymes (in addition to p300/CBP) are involved, and how these enzymes coordinate with the aforementioned transcriptional regulators.

One determinant for chromatin structure and functional state is histone methylation that occurs on specific lysine residues in histones [12], [13]. Five lysine residues within the N-terminal tail of histone H3 (K4, K9, K27, and K36) and H4 (K20) have been shown to be the sites for methylation. These lysine residues can be mono-, di-, or trimethylated. Depending on the specific lysine residues and the degree of methylation, histone methylation can have distinct effects on gene expression. In general, histone H3K4 and K36 di-and trimethylation, and H3K27 monomethylation are associated with actively transcribed genes, whereas H3K9 and K27 di- and trimethylation are considered repressive markers for gene expression. The distribution pattern of histone methylation on gene loci can also be quite different. For example, H3K4 and K9 methylation are enriched in the promoter regions, whereas K36 di- and trimethylation are mainly located in the coding regions and their levels peak toward the 3′end of the gene [14]–[16]. By altering chromatin structure, histone methylation fine-tunes transcriptional outputs.

Histone methylation is reversible and its dynamic nature is controlled by a balance between histone methyltransferases and histone demethylases. A number of histone demethylases have been identified in recent years and they are classified into two groups [17]–[20]. The first group contains two genes, LSD1 and LSD2, in human genome. These enzymes catalyze demethylation via an FAD-dependent oxidative reaction that requires protonated nitrogen in the substrate [19]. The second group are genes that contain a JmjC domain. Nineteen members of the JmjC domain-containing proteins in the human genome have been shown to be demethylases. The JmjC domain is the catalytic domain that possesses demethylation activity. These enzymes use Fe(II) and the intermediate metabolite α-ketoglutarate as co-factors to catalyze a hydroxylation-based demethylation [20]. Because of their enzymatic requirement for either FAD or α-ketoglutarate, it has been postulated that histone demethylases might be important for energy homeostasis by linking metabolic signals to chromatin status and transcriptional regulation [21]. Here, through both in vitro and in vivo studies, we reveal an important regulatory function of histone demethylase Jhdm1a in gluconeogenesis that is mediated by its active demethylation on the C/EBPα locus.

## Results

### Identification of the histone demethylase Jhdm1a as a negative regulator of gluconeogenic gene expression

**Figure 1:**
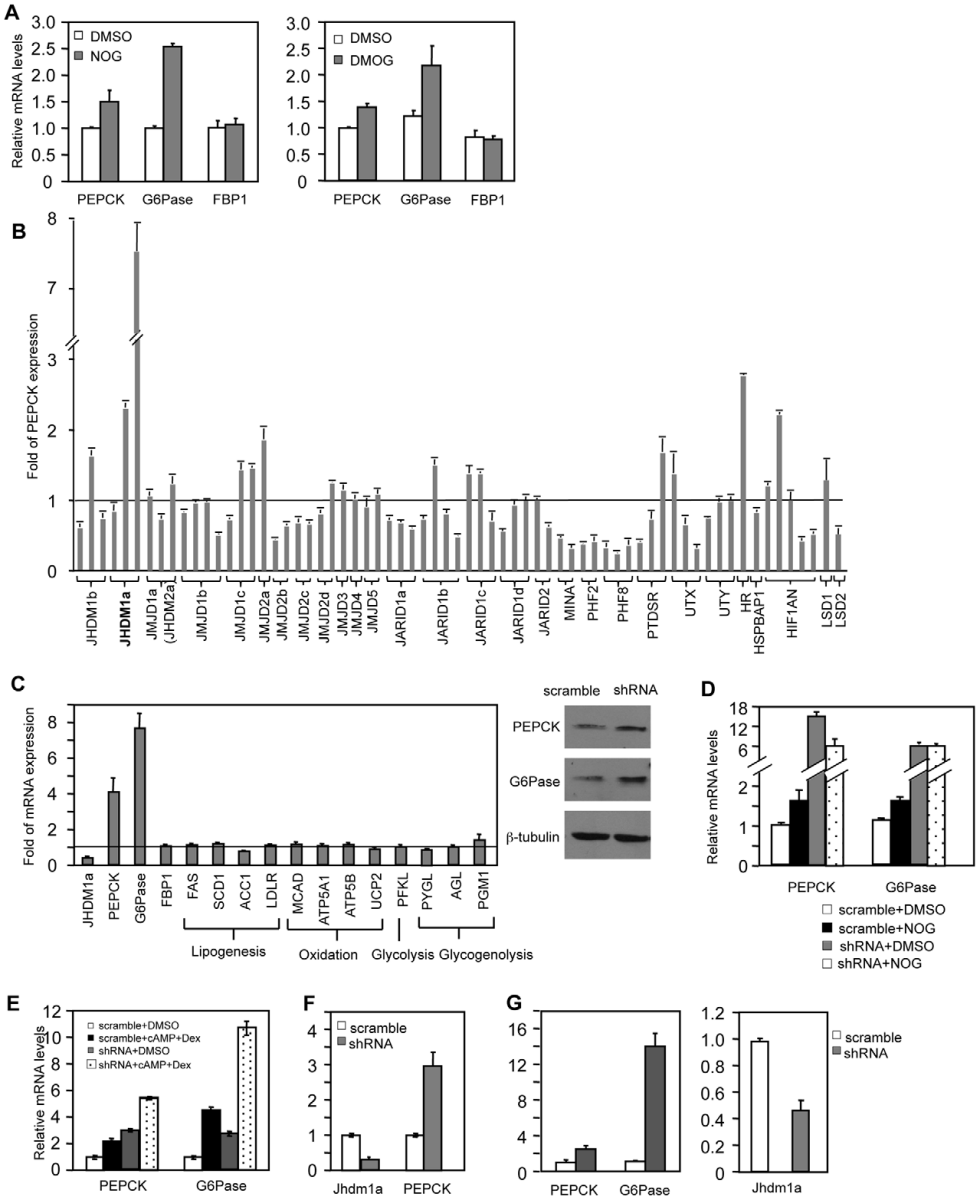
Knockdown of Jhdm1a specifically upregulates PEPCK and G6Pase expression in cultured hepatic cells. (A) HepG2 cells were treated with DMSO, NOG (1 mM), or DMOG (0.1 mM) for 12 hr. (B) shRNA-mediated screen. Each bar represents a single shRNA construct. Data were presented as fold relative to the scramble control. (C) Jhdm1a was knocked down in HepG2 cells with shRNA lentiviruses. Left, gene expression data were presented as fold relative to the scramble control from three experiments. Full names of individual genes are listed in Table S1. Right, levels of PEPCK and G6Pase protein were determined. (D) Jhdm1a knockdown or scramble HepG2 cells were treated with NOG (1 mM) for 12 hr. (E) Jhdm1a knockdown or scramble HepG2 cells were treated with a combination of dibutyryl cyclic-AMP (cAMP, 0.5 mM) and dexamethasone (Dex, 1 µM) in DMEM medium for 6 hr. Data were from two experiments. (F) Lentiviral knockdown of Jhdm1a in mouse hepatoma HepA1-6 cells. Data were from two experiments. (G) Adenoviral knockdown of Jhdm1a in mouse primary hepatocytes. Experiments were repeated three times with similar results.

To assess whether JmjC domain-containing histone demethylase(s) is involved in the regulation of gluconeogenesis, we treated human hepatoma HepG2 cells with N-oxalylglycine (NOG) or its derivative, dimethyloxalylglycine (DMOG), and examined the expression of the gluconeogenic enzymes. NOG and DMOG are analogues of α-ketoglutarate and are general enzymatic inhibitors of the JmjC domain-containing histone demethylases [22], [23]. Treatment with either compound led to an increase of PEPCK and G6Pase expression (Figure 1A), indicating a potential requirement of histone demethylation activity in the regulation of gluconeogenesis.

We next decided to use shRNA knockdown to identify the involved histone demethylase(s). As both the hormonal and molecular pathways that regulate PEPCK and G6Pase transcription are retained in HepG2 cells, we performed our screening experiments in these cells. We obtained a collection of human lentiviral shRNA constructs against the known JmjC domain-containing demethylases and a few JmjC domain-containing proteins where an enzymatic function has not been ascribed. We also included shRNA constructs against the FAD-dependent histone demethylases (LSD1 and LSD2). We stably expressed individual knockdown constructs in HepG2 cells and screened by quantitative RT-PCR for an increase of PEPCK expression compared with scramble controls. We found that knockdown of the JmjC-domain-containing protein Jhdm1a had the strongest effect (Figure 1B). Jhdm1a is a histone demethylase that specifically demethylates dimethylated H3K36 [20]. Knockdown of this demethylase also robustly promoted G6Pase expression, but not FBP-1 expression (Figure 1C). Not surprisingly, this led to an increase of PEPCK and G6Pase protein levels (Figure 1C). It is likely that NOG-induced PEPCK and G6Pase expression is mediated through inhibition of Jhdm1a, as the induction was lost in Jhdm1a knockdown cells (Figure 1D). As expected, treatment of HepG2 cells with dibutyryl cyclic-AMP and dexamethasone stimulated PEPCK and G6Pase expression; knockdown of Jhdm1a further led to an additive/synergistic increase, indicating a possibility that the effect of Jhdm1a knockdown is independent of the pathway activated by the hormones (Figure 1E). Similar results were obtained in HepG2 cells with an independent Jhdm1a silencing construct (Figure S1). In addition, we generated a lentiviral knockdown construct that targeted mouse Jhdm1a and expressed it in mouse hepatoma HepA1-6 cells. These cells express low level of PEPCK and undetectable level of G6Pase. Silencing of Jhdm1a in these cells elevated PEPCK expression (Figure 1F), while expression of key gluconeogenic transcriptional regulator Foxo1 and PGC-1α was not increased (Figure S2). We next studied gluconeogenic gene expression in a more physiological setting. We knocked down Jhdm1a in primary mouse hepatocytes using adenovirus and found that PEPCK and G6Pase expression was increased as well (Figure 1G). These results collectively demonstrated a negative role of Jhdm1a in gluconeogenic gene expression. Finally, we determined whether Jhdm1a regulates other metabolic pathways in HepG2 cells. We found that knockdown of Jhdm1a did not affect expression of any examined genes involved in lipogenesis, fatty acid oxidation, glycolysis, or glycogenolysis (Figure 1C), suggesting a quite specific metabolic function of Jhdm1a.

### Ectopic expression of Jhdm1a suppresses gluconeogenic gene expression in a demethylation activity-dependent manner

**Figure 2:**
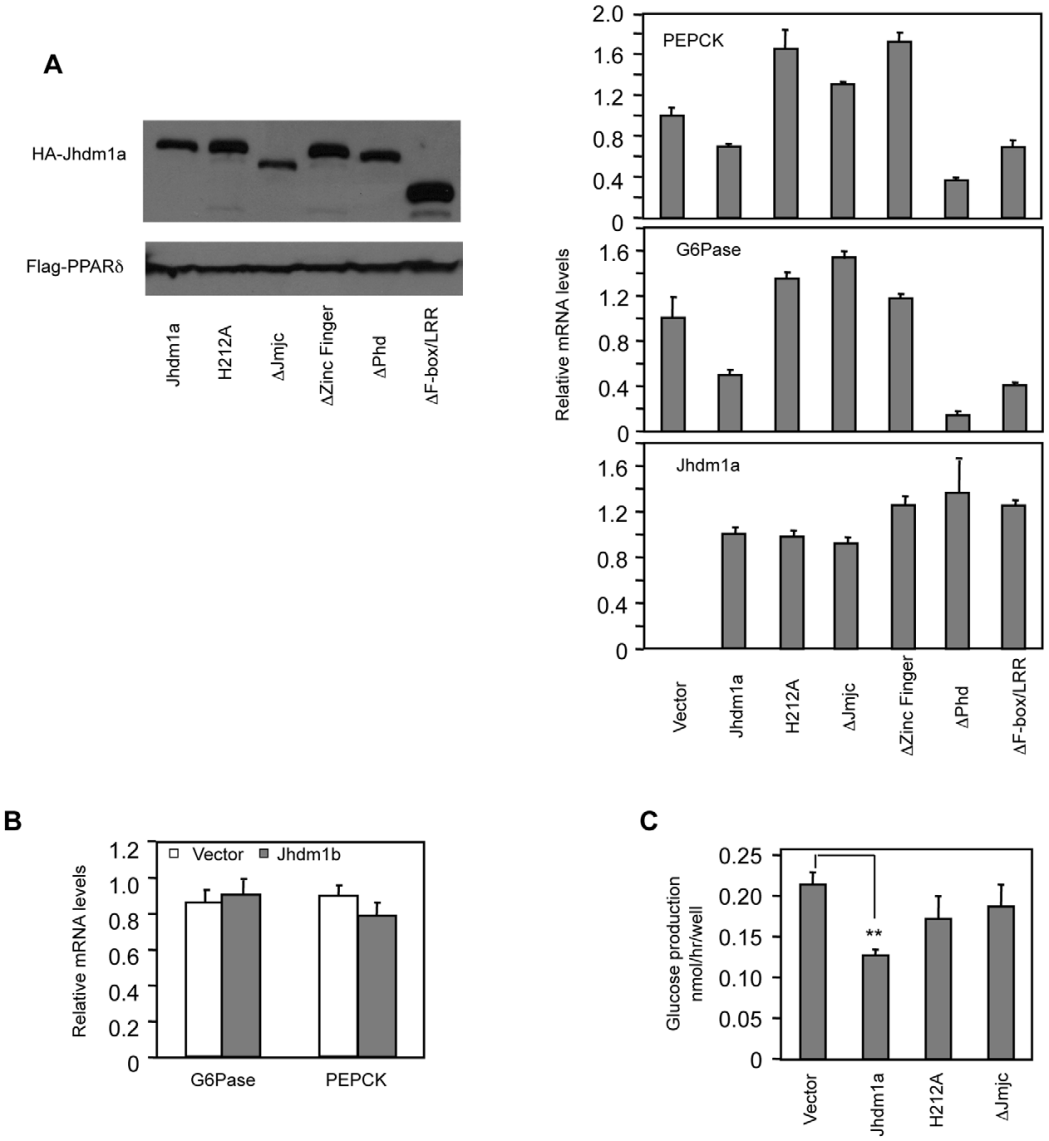
Suppression of gluconeogenic gene expression by Jhdm1a requires its demethylation activity. (A) (Left panel) Jhdm1a constructs were tagged with HA and transfected into Hela cells to ensure they express stable proteins. Co-transfected Flag-PPARδ serves as transfection and loading control. (Right, three panels) Jhdm1a constructs were lentivirally expressed in HepG2 cells. Data were shown from one representative of five experiments with similar results. (B) Lentiviral expression of Jhdm1b does not suppress gluconeogenic gene expression. (C) Jhdm1a constructs were lentivirally expressed in rat hepatic FAO cells and glucose production was measured. Data were from triplicates. **, P<0.01.

Given that knockdown of Jhdm1a elevates gluconeogenic gene expression, we examined whether an opposite effect could be observed in cells expressing Jhdm1a. We stably expressed Jhdm1a via lentivirus in liver cells and found that this expression decreased both basal and hormonal-stimulated levels of PEPCK and G6Pase mRNA (Figure 2A and Figure S3). Interestingly, and in agreement with the knockdown data (Figure 1B), ectopic expression of demethylase Jhdm1b, which is closely related to Jhdm1a, did not inhibit gluconeogenic gene expression (Figure 2B and Figure S4). To determine the domains in Jhdm1a that are required for its suppressive function, we generated a series of Jhdm1a mutants. We first confirmed that these mutants were capable of producing stable proteins at a similar level, as judged by plasmid transfection in Hela cells (Figure 2A). We then expressed the mutants in HepG2 cells through lentivirus with a similar, low infection efficiency. Deletion of the JmjC domain or the CXXC Zinc finger domain abolished the suppression on PEPCK and G6Pase expression, whereas mutant lacking either the PHD domain or the F-box and Leucine-rich repeats remained fully functional (Figure 2A and Figure S3). Note that these Jhdm1a mutants were expressed at similar mRNA levels as their wild-type counterpart. The JmjC domain harbors the histone demethylation activity. Consistent with the effect of the JmjC deletion mutant, a demethylation-dead point mutant (H212A) [20] of Jhdm1a was no longer able to suppress PEPCK and G6Pase expression (Figure 2A and Figure S3). We next determined the effect of Jhdm1a on glucose production in vitro. We found that ectopic expression of wild type Jhdm1a, but not the demethylation defective mutants, inhibited glucose production in rat hepatoma FAO cells (Figure 2C). Taken together, these results demonstrate that both the demethylation activity and the CXXC Zinc finger domain of Jhdm1a are required for its negative modulation of gluconeogenic gene expression.

### Jhdm1a regulates gluconeogenesis in vivo

**Figure 3:**
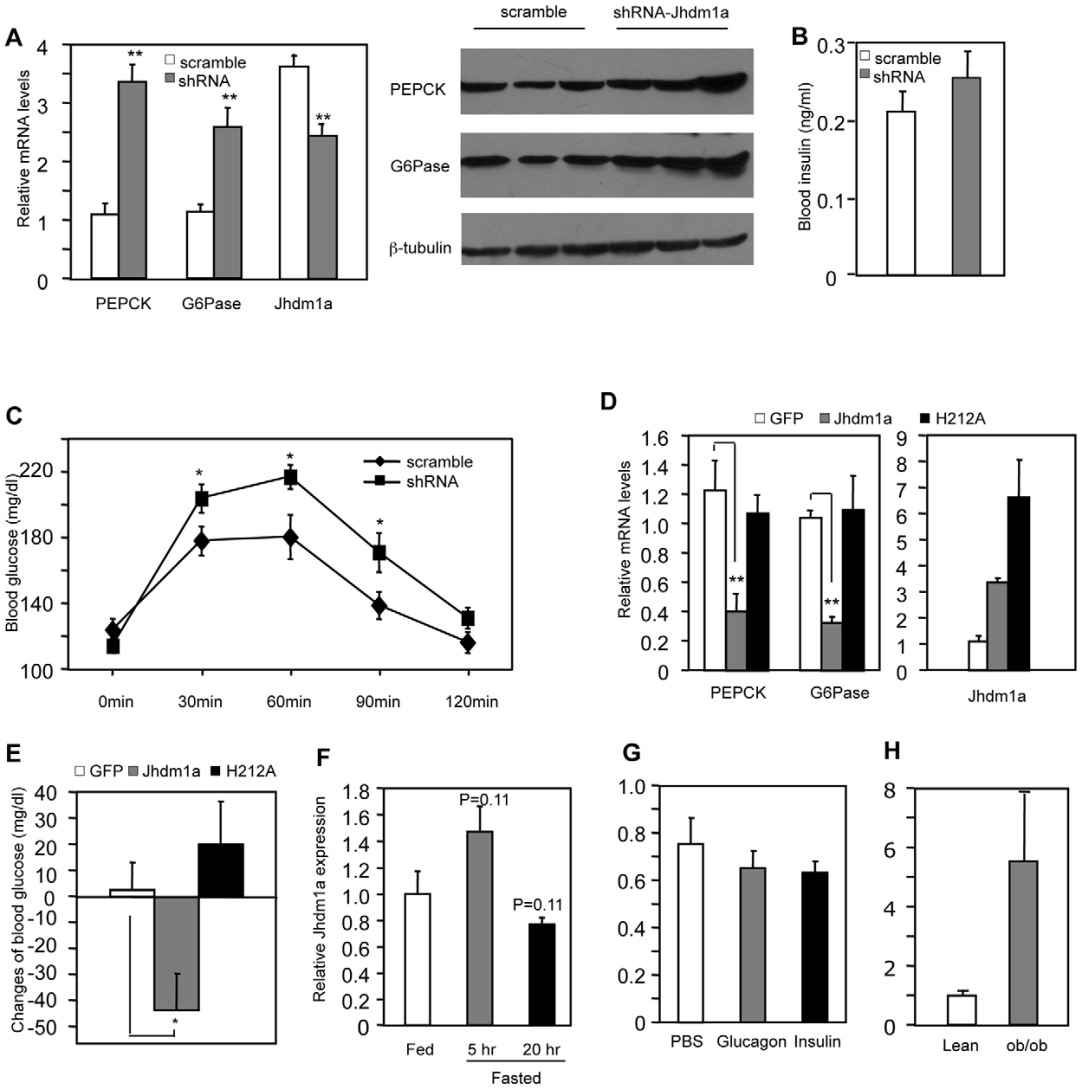
Jhdm1a regulates gluconeogenesis in vivo. (A) Jhdm1a knockdown or scramble adenoviruses were transduced into the liver of wild-type male C57BL/6J mice (n = 5 per group). Mice fed *ad libitum* were sacrificed at Day 5 after viral infusion. (Left) mRNA levels of PEPCK, G6Pase and Jhdm1a in the liver were measured and normalized to U36b4. **, P<0.005. (Right) PEPCK and G6Pase protein. (B) Blood insulin levels at fed state were measured at Day 5. (C) Jhdm1a knockdown or scramble adenoviruses were transduced into the liver of wild-type male C57BL/6J mice (n = 10 per group). At Day 5, mice were i.p. injected with pyruvate (2 g/kg body weight) after a starvation for 16 hr and blood glucose levels were measured. *, P<0.05. (D and E) Adenoviruses expressing wild-type Jhdm1a, H212A point mutant, or GFP were transduced into the liver of male *ob/ob* mice (n = 5 per group). Gene expression was measured on Day 5 and blood glucose levels were measured on Day 3 after a 5-hr fasting. Changes of blood glucose level relative to Day 0 are presented. *, P<0.03; **, P<0.01. (**F**) Hepatic Jhdm1a mRNA levels in male C57BL/6J mice (n = 5 per group) fed *ad libitum*, or fasted for 5 hr or 20 hr. (G) Male C57BL/6J mice (n = 4) were i.p. injected with glucagon (300 µg/kg), insulin (0.75 U/Kg), or PBS. Hepatic Jhdm1a mRNA levels were examined 6 hr after injection. (H) Hepatic Jhdm1a mRNA levels in lean mice and diabetic *ob/ob* mice (n = 3 per group).

Based on our discovery of the regulatory role of Jhdm1a in vitro, we tested whether Jhdm1a regulates gluconeogenesis in live animals. We obtained five lentiviral Jhdm1a knockdown constructs from Open Biosystems and tested their knockdown efficiency by RT-QPCR in mouse cell culture. We transferred two best ones into an adenoviral vector, generated adenoviruses, and further confirmed that they were able to reduce ectopically expressed Jhdm1a protein level in vitro (Figure S5). The viruses were infused into the liver of wild-type C57BL/6J mice via tail vein injection and endogenous Jhdm1a expression was decreased, which led to a significantly increase in hepatic expression of PEPCK and G6Pase in both fed and fasting states, compared with the scramble control (Figure 3A, Figure S6 and S7). A corresponding enhanced PEPCK and G6Pase protein production was observed (Figure 3A). Blood insulin levels examined at fed state were not significantly different (Figure 3B). Although the Jhdm1a knockdown mice were still able to maintain normal glycemia, they displayed higher glucose production upon injection of the gluconeogenic substrate pyruvate (Figure 3C). We next ectopically expressed either wild-type Jhdm1a or the H212A point mutant in the liver of diabetic *ob/ob* mice. Expression of the wild-type Jhdm1a, but not the H212A point mutant, decreased the expression of PEPCK and G6Pase (Figure 3D). Accordingly, we observed a statistically significant reduction of blood glucose level in *ob/ob* mice expressing wild-type Jhdm1a (Figure 3E). Thus, Jhdm1a indeed has a physiological role in hepatic gluconeogenesis in vivo, and this role is mediated by its histone demethylation activity.

Gluconeogenesis is activated during fasting and suppressed by a meal. Interestingly, the hepatic expression Jhdm1a was not changed during either a short-fasting (5 hr) or a long-fasting (20 hr) (Figure 3F). Furthermore, administration of either glucagon or insulin in vivo revealed no difference in Jhdm1a expression (Figure 3G). Likewise, treatment of HepG2 cells with dibutyryl cyclic-AMP and dexamethasone or insulin had no effect on Jhdm1a expression (Figure S8). Although we cannot rule out the possibility of post-transcriptional regulation of Jhdm1a by hormonal signaling, these data, together with the observed effects of Jhdm1a on PEPCK and G6Pase expression in both non-stimulatory and stimulatory conditions (Figure 1C and 1E, Figure 3A, and Figures S6 and S7), indicate that Jhdm1a acts as a negative regulatory mechanism to fine-tune baseline gluconeogenesis. In diabetic *ob/ob* mice, Jhdm1a expression was elevated (Figure 3H), possibly reflecting a feedback response.

### Regulation of gluconeogenesis by Jhdm1a is mediated through C/EBPα expression

**Figure 4:**
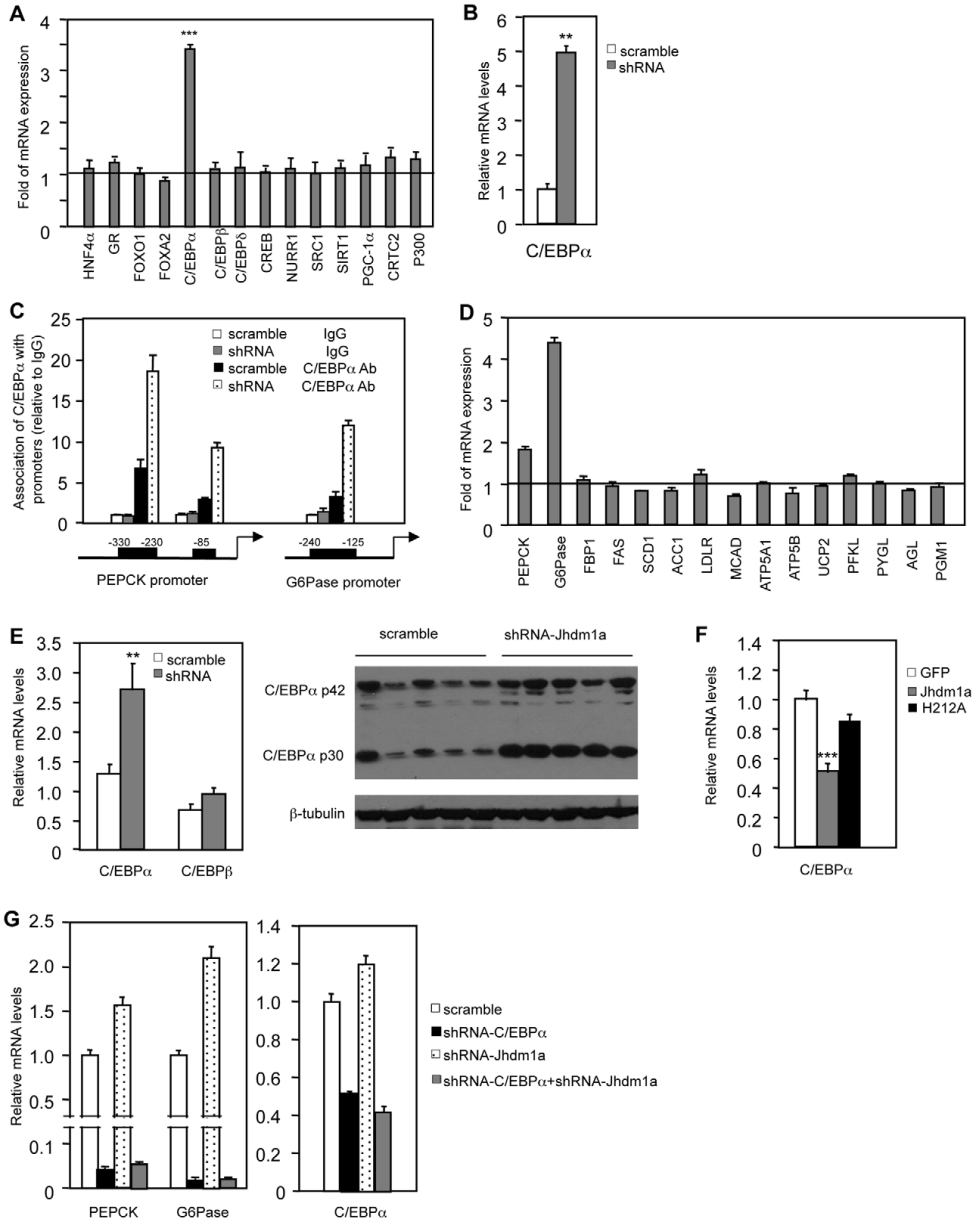
Jhdm1a regulates the expression of C/EBPα, thereby indirectly modulating gluconeogenic gene expression. (A) Jhdm1a was knocked down in HepG2 cells with shRNA lentiviruses. Expression levels of known transcriptional regulators for gluconeogenesis were examined. Data are presented as fold relative to the scramble control from three experiments. ***, P<0.00005. (B) C/EBPα expression in Jhdm1a knockdown mouse primary hepatocytes. (C) Jhdm1a was knocked down in HepG2 cells with shRNA lentiviruses. Endogenous C/EBPα association with known binding sites on the PEPCK and G6Pase promoters was examined by ChIP assay. (D) Gene expression in HepG2 cells infected with lentiviruses expressing C/EBPα or vector. (E) Increased C/EBPα expression in the liver of wild-type C57BL/6J mice (n = 5 per group) with Jhdm1a knockdown. C/EBPα mRNA level and protein level were shown from independent groups of mice. **, P<0.02. (F) Decreased C/EBPα expression in the liver of *ob/ob* mice (n = 5 per group) ectopically expressing wild-type Jhdm1a, but not in the liver expressing H212A point mutant. ***, P<0.001. (G) HepG2 cells were infected with lentiviruses expressing C/EBPα shRNA and selected with puromycine. Cells were then infected with lentiviruses expressing Jhdm1a shRNA without selection. Data were shown from one representative of four experiments. Note, the low induction of PEPCK and G6Pase expression by Jhdm1a knockdown is due to the lack of selection pressure.

We explored how Jhdm1a regulates gluconeogenesis. We initially speculated that Jhdm1a might associate with the transcriptional regulator complex on the promoters of PEPCK and G6Pase and directly regulate their expression. To test this idea, we performed chromatin immunoprecipitation experiments in HepG2 cells ectopically expressing HA-tagged Jhdm1a. Unexpectedly, Jhdm1a did not associate with either PEPCK promoter or G6Pase promoter (Figure S9). The promoter regions we examined have been well characterized previously and are subjected to extensive regulation by an array of transcription regulators [6]–[9]. The lack of association of Jhdm1a with PEPCK and G6Pase promoters indicates to us that Jhdm1a might not directly regulate the expression of these two genes. We thus considered a possibility that Jhdm1a instead regulates the expression of any of the involved transcription factors or co-factors [10], [11]. We knocked down Jhdm1a in HepG2 cells and examined their expression. We found that the transcription factor C/EBPα was the only one whose expression level was significantly increased (Figure 4A). Similarly, knockdown of Jhdm1a promoted C/EBPα expression in primary mouse hepatocytes (Figure 4B). As a result of increased C/EBPα level, the association of C/EBPα with its binding sites within the PEPCK and G6Pase promoters was strongly enhanced in Jhdm1a knockdown HepG2 cells (Figure 4C). Members of C/EBPs were shown to activate the expression of PEPCK and G6Pase in vitro [24], [25]. We confirmed these previous results and also observed a remarkably similar target gene expression pattern between Jhdm1a silencing and C/EBPα ectopic expression (comparing Figure 1C and Figure 4D), supporting a functional connection between Jhdm1a and C/EBPα.

Previous work by others has also demonstrated an essential in vivo role of C/EBPα in hepatic PEPCK and G6Pase expression [26]–[29]. Importantly, we found that in vivo knockdown of Jhdm1a in the mouse liver increased the level of C/EBPα (Figure 4E and Figure S10). Conversely, exogenous expression of Jhdm1a in the liver suppressed C/EBPα expression, whereas the H212A point mutant had no effect (Figure 4F). To further examine whether the action of Jhdm1a is C/EBPα-dependent, we knocked down both Jhdm1a and C/EBPα in hepatic cells. The increase of PEPCK and G6Pase expression caused by Jhdm1a knockdown was greatly diminished in the double knockdown cells (Figure 4G). The results together suggest that Jhdm1a regulates gluconeogenesis, at least in part, through its control of C/EBPα expression. As previously noted [11], C/EBPα expression remained unchanged during both short and long fasting (data not shown), in agreement with our observation that Jhdm1a expression was not affected by these conditions.

### USF1 mediates the recruitment of Jhdm1a to the C/EBPα promoter

**Figure 5:**
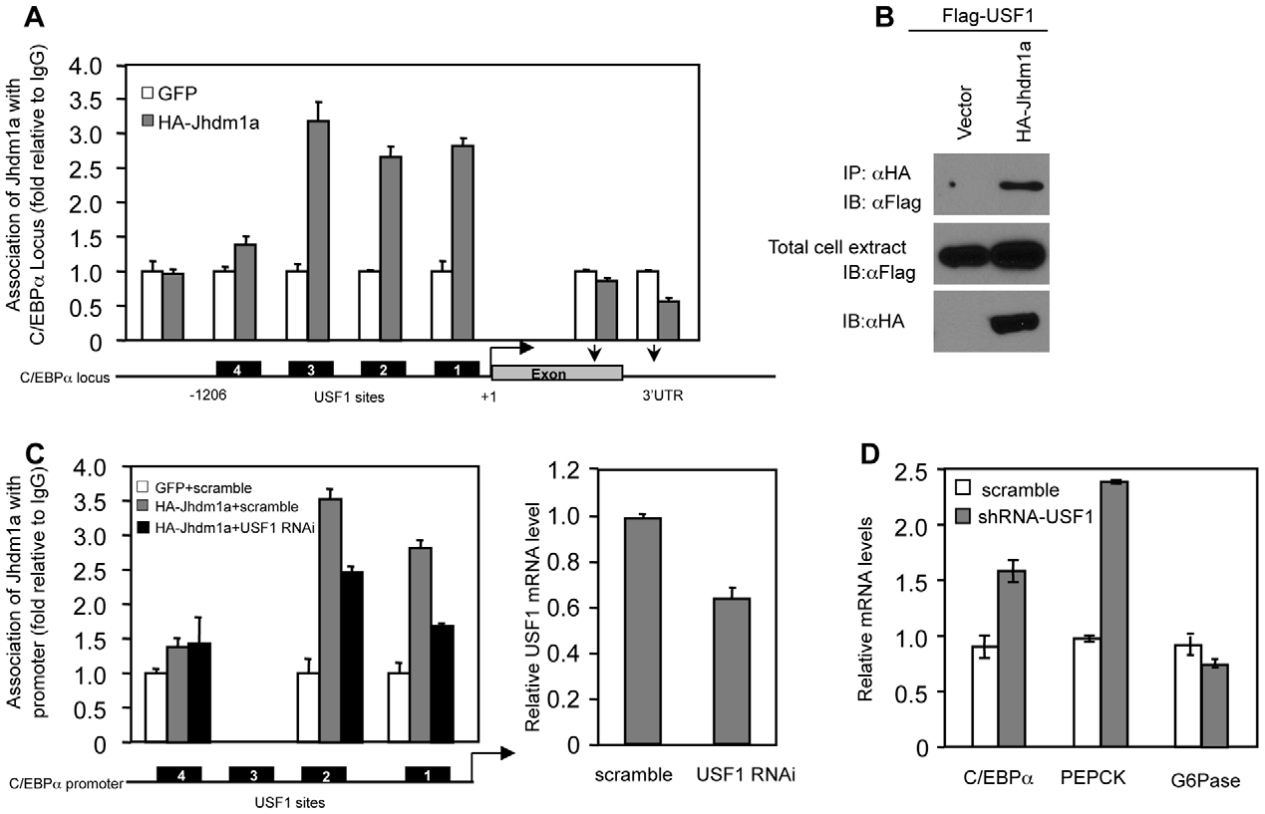
Suppression of C/EBPα expression by Jhdm1a is mediated by USF1. (A) Jhdm1a associates with putative USF1-binding sites on the C/EBPα promoter region. Adenoviral HA-Jhdm1a were expressed in HepG2 cells and ChIP assays were performed with HA antibody. Data were shown from one representative of three experiments with similar results. (B) Jhdm1a interacts with USF1. Hela cells were co-transfected with indicated plasmids. Cell extracts were incubated with HA beads and immunoprecipitates were probed with Flag antibody. (C) HA-Jhdm1a (adenoviral) along with shRNA (lentiviral) against USF1 was co-expressed in HepG2 cells. ChIP assays were performed with HA antibody. (D) Gene expression in HepG2 cells expressing lentiviral USF1 shRNA. Data were shown from one representative of three experiments.

To identify the molecular mechanism by which Jhdm1a regulates C/EBPα expression, we first examined whether Jhdm1a associates with the C/EBPα locus. The C/EBPα locus contains a single exon. We expressed HA-tagged Jhdm1a in hepatic cells and performed chromatin immunoprecipitation experiments using antibody against the HA tag. We found that Jhdm1a was associated with the C/EBPα promoter region but not with the intragenic region (Figure 5A). Interestingly, this promoter region contains four separate transcription factor USF1 binding sites that have been implicated in C/EBPα expression [30], [31] and Jhdm1a was present on three of them. An interaction between Jhdm1a and USF1 was readily detected in cells expressing both of them (Figure 5B). Moreover, reduction of USF1 level by shRNA-mediated silencing diminished the association of exogenous Jhdm1a with these sites (Figure 5C). Despite the high background of the Jhdm1a antibody, we were also able to show that endogenous Jhdm1a associated with the USF1 binding sites, since knockdown of Jhdm1a decreased its association with these sites (Figure S11). Functionally, knockdown of USF1 led to an increase of C/EBPα expression and accordingly, an increase of PEPCK expression (Figure 5D). These data suggest a model in which USF1 recruits Jhdm1a to the C/EBPα promoter to negatively regulate its expression.

### Jhdm1a actively demethylates dimethylated H3K36 on the C/EBPα locus

**Figure 6:**
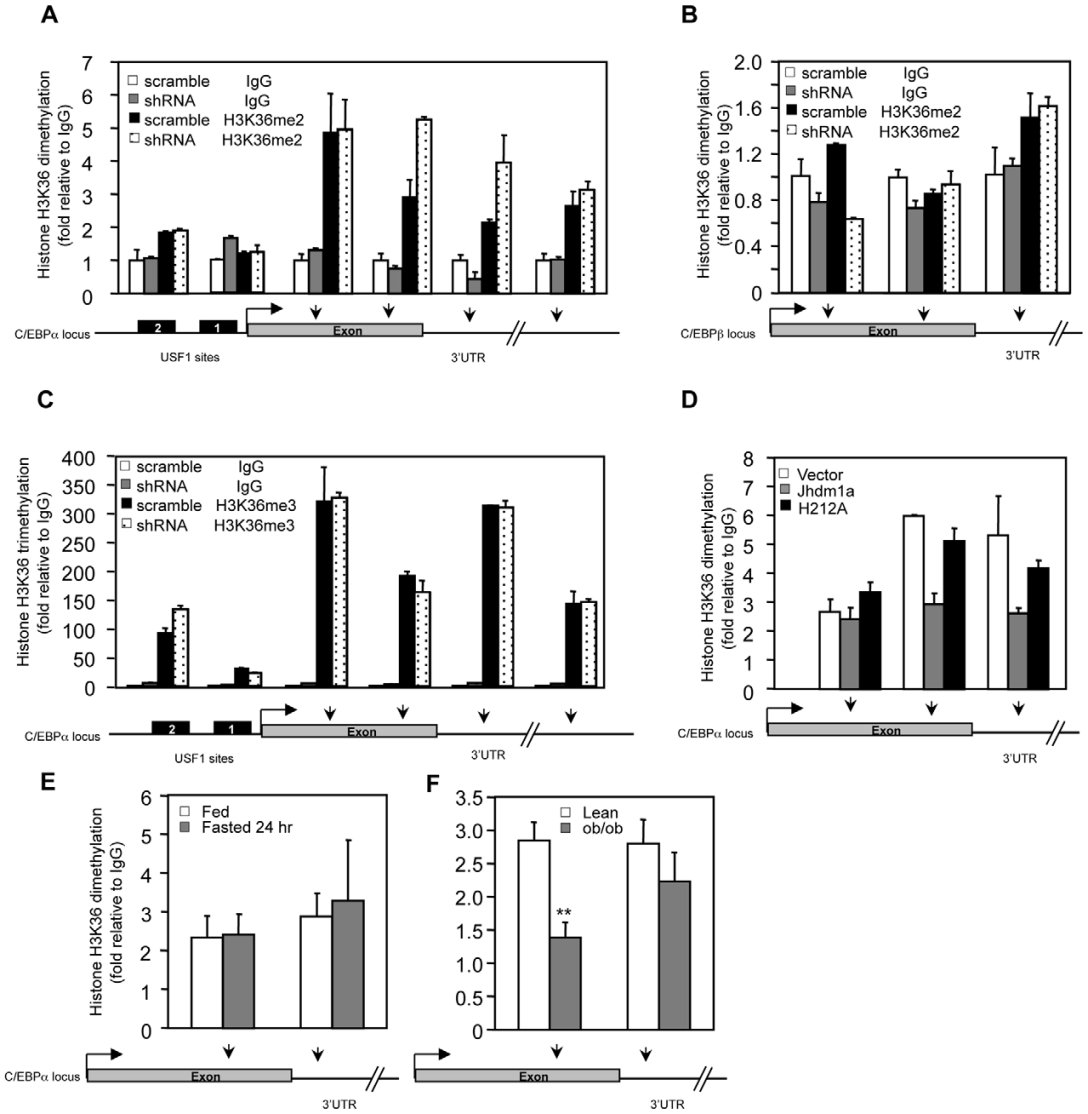
Jhdm1a specifically demethylates dimethylated H3K36 on the C/EBPα locus. (A) Knockdown of Jhdm1a increases H3K36 dimethylation (H3K36me2) on the C/EBPα locus. Data were shown from one representative of three independent experiments with similar results. (B) Knockdown of Jhdm1a does not affect H3K36me2 on the C/EBPβ locus. (C) Knockdown of Jhdm1a does not increase H3K36 trimethylation (H3K36me3) on the C/EBPα locus. (D) Wild-type Jhdm1a, but not the H212A point mutant, decreases H3K36me2 on the C/EBPα locus. (E) H3K36me2 on the C/EBPα locus is not modulated by feeding/fasting conditions. n = 4 per group. (F) Decreased H3K36me2 on the C/EBPα locus in *ob/ob* mice. n = 4 per group. **, P<0.01.

Given the association of Jhdm1a with the C/EBPα promoter, we examined whether Jhdm1a modulates the H3K36 methylation pattern on the C/EBPα locus. Knockdown of Jhdm1a increased H3K36 dimethylation in the 3′ exon region and 3′ UTR that is close to the exon, but had little effect on H3K36 dimethylation on the promoter, 5′ exon region, and 3′UTR that is located far away from the exon (Figure 6A). This pattern of modulation is in concord with the previously shown genome-wide distribution of H3K36 dimethylation where it is mostly found in the intragenic region and usually peaks toward 3′ exon [15]. The demethylation by Jhdm1a is gene-specific, as knockdown of Jhdm1a did not increase H3K36 dimethylation at the C/EBPβ locus (Figure 6B). Moreover, knockdown of Jhdm1a did not affect the H3K36 trimethylation pattern at the C/EBPα locus (Figure 6C), consistent with the enzymatic property of Jhdm1a to specifically demethylate dimethylated H3K36 [20]. Next, we examined the effect of ectopically expressed Jhdm1a on H3K36 dimethylation at the C/EBPα locus. We found that expression of wild type Jhdm1a, but not of the H212A mutant, led to a significant decrease of K36 dimethylation (Figure 6D). These results suggest that Jhdm1a demethylates dimethylated H3K36 at the C/EBPα locus, hence directly regulating its expression.

We determined whether the H3K36 dimethylation status at the C/EBPα locus is modulated by hormonal signaling or metabolic states. In agreement with Jhdm1a expression (Figure 3F and 3G, and Figure S8), we found that levels of H3K36 dimethylation remained unchanged in HepG2 cells treated with hormones (Figure S12) or in livers of fasted mice (Figure 6E), supporting the idea that Jhdm1a and H3K36 dimethylation at the C/EBPα locus are primarily involved in basal control of gluconeogenesis. Interestingly, H3K36 dimethylation was significantly decreased at the exon region of C/EBPα locus in diabetic *ob/ob* mice (Figure 6F), likely due to increased Jhdm1a expression (Figure 3H). These data suggest a possible physiological, compensatory attempt to suppress hyperglycemia in *ob/ob* mice.

## Discussion

In recent years, a number of histone demethylases have been identified [17]–[20]. While these exciting discoveries dramatically reversed our previous view that histone methylation was a stable, non-erasable marker, our knowledge regarding the functions of these demethylases in biological processes and diseases is very limited. Here, through an shRNA screen against the known histone demethylases, we identify Jhdm1a negatively regulates gluconeogenic gene PEPCK and G6Pase expression both in vitro and in vivo. Phenotypically, silencing of Jhdm1a elevates glucose production, whereas its ectopic expression lowers blood glucose levels in diabetes. Interestingly, our studies suggest that Jhdm1a does not appear to control PEPCK and G6Pase expression directly. Rather, Jhdm1a exerts its function through C/EBPα. The role of C/EBPα in gluconeogenesis has been well established [25]–[29]. We found that Jhdm1a negatively modulates the expression of C/EBPα through active demethylation on the C/EBPα locus. Therefore, our work potentially uncovers a novel molecular mechanism in gluconeogenesis, where histone demethylation regulates a key gluconeogenic transcription factor. However, it is important to note that our in vivo studies were performed using adenoviral infusion to acutely manipulate hepatic Jhdm1a level, therefore, chronic and more physiological and pathophysiological roles of Jhdm1a in gluconeogenesis remain to be addressed in detail with liver-specific Jhdm1a knockout and transgenic models. In addition, as genetic variations at the Jhdm1a locus are present in human population, it will be interesting to analyze whether these variations are associated with type 2 diabetes.

It was hypothesized that histone demethylases might be important for metabolic homeostasis [21]. This is supported by the obese phenotype of mice deficient for H3K9 histone demethylase, Jhdm2a [32], [33]. Our demonstration of Jhdm1a functioning in gluconeogenesis provides another example. It is anticipated that future studies will reveal additional histone demethylases as important regulators of energy metabolism. Histone demethylases are considered as global modifiers of chromatin structure, however, it is clear that a particular demethylase only regulates a small subset of genes and therefore, a specific metabolic pathway. This specificity is likely to be determined by the target tissue, the repertoire of transcriptional regulators in that tissue, and whether this particular demethylation on individual gene locus is sufficient to translate into a gene expression readout.

Histone H3K36 di- and trimethylation have been shown to be associated with actively transcribed genes and their levels peak near the 3′ end of the gene [14]–[16], [34]. In yeast, K36 di- and trimethylation have been implicated in transcriptional elongation by preventing cryptic, intragenic transcription [35]–[37]. In higher eukaryotes, the exact function of K36 methylation is poorly understood. We show here that Jhdm1a demethylates dimethylated H3K36 on the C/EBPα locus and negatively regulates its expression. Although we cannot rule out the possibility that changes of dimethylated H3K36 level are secondary due to C/EBPα expression, the requirement for the demethylase activity of Jhdm1a and the unaffected H3K36 trimethylation on the C/EBPα locus strongly argue that this is unlikely. Jhdm1b is another demethylase that targets dimethylated H3K36. Jhdm1b-mediated demethylation was recently shown to negatively regulate the expression of the p15^Ink4b^ tumor suppressor [38]. These studies suggest a positive role of H3K36 dimethylation for gene expression.

Our data suggest that Jhdm1a is recruited by USF1 to the USF1-binding sites within the C/EBPα promoter. A recent study shows that Jhdm1a, through its CXXC Zinc finger domain, associates with unmethylated CpG islands on gene promoters [39]. Indeed, the C/EBPα promoter is considerably CpG-rich, and we find that the CXXC Zinc finger domain is required for the suppressive function of Jhdm1a. Therefore, it is possible that the CpG-rich sequences and USF1 cooperatively mediate the recruitment of Jhdm1a. One interesting observation in our study is that Jhdm1a demethylates C/EBPα intragenic region that lacks detectable association. It is possible that the initial recruitment by USF1 to the promoter is a relatively stable state, but following recruitment, Jhdm1a moves along the gene body to demethylate dimethylated H3K36. Thus the association of Jhdm1a with the gene body might be transient and difficult to capture. There are precedents of similar observations. For example, ChIP-seq studies reveal that, for actively transcribed genes, Pol II is predominantly detected at transcription start sites, not transcribed regions [16]. PHF8, a H4K20/H3K9 demethylase, was found to demethylate regions that it does not associate with [40]. Clearly, how epigenetic enzymes are recruited and are able to modify chromatin structure in a widespread fashion is a fascinating question to be fully understood.

While Jhdm1a-catalyzed histone demethylation regulates gluconeogenesis through an indirect mechanism by targeting C/EBPα, a previous report has postulated that dimethylation of histone H3 arginine 17 has a direct impact on gluconeogenic gene expression, as the level of this modification on the PEPCK promoter increases with dexamethasone treatment and decreases upon subsequent addition of insulin [41]. However, the molecular events responsible for and the functional outcome of this change were unknown. Nevertheless, their studies, along with ours, indicate that histone methylation/demethylation could be more commonly employed than we appreciated to regulate gluconeogenesis at multiple layers. To our surprise, Jhdm1a expression, hence the H3K36 dimethylation status at the C/EBPα locus, are not influenced by fed and fasted states and hormonal signaling. Our data indicate that, under normal conditions, Jhdm1a-mediated demethylation primarily function in maintaining basal-state gluconeogenesis irrespective of nutritional and hormonal cues. In support of this model, we found that knockdown of Jhdm1a in mice elevates the expression of C/EBPα, PEPCK and G6Pase in both fed and fasted states. Mechanisms controlling hormonal-regulated gluconeogenesis have been extensively studies [11], less was understood for basal-state gluconeogenesis. Our work provides insights into this key process. Interestingly, in diabetic state, Jhdm1a expression is increased and H3K36 dimethylation at the C/EBPα locus is decreased, indicating a possible involvement of Jhdm1a in counteracting hyperglycemia. Thus, under pathophysiological conditions such as obesity and insulin resistance, the expression and/or activity of Jhdm1a can be modulated by currently unknown mechanisms. In addition, we find that in fetal liver, Jhdm1a is highly expressed and C/EBPα level is very low; in neonatal stage, hepatic Jhdm1a level decreases and C/EBPα level increases (our unpublished data). As gluconeogenesis occurs in neonatal stage but not in embryonic stage, whether Jhdm1a is involved in this metabolic transition during development remains to be determined. In summary, our results illustrate how the dynamics of H3K36 dimethylation regulates basal gluconeogenesis and indicate that increasing the demethylation activity of Jhdm1a could potentially offer therapeutic benefits to curb hyperglycemia.

## Materials and Methods

### Lentiviral knockdown

Lentiviral shRNA constructs (pGIPZ-based; Open Biosystems) against the known human demethylases were obtained through the RNAi Core Facility at University of Massachusetts Medical School. All other lentiviral shRNA constructs were obtained directly from Open Biosystems. All relevant constructs were verified and their targeting sequences are provided in Table S1. Lentiviruses were packaged as described [42]. After virus infection, cells were re-plated next day and selected with puromysin for three days. Cells were then trypsinized and plated at a similar confluency. Cells were cultured in the presence of puromycin for two more days and total RNA was isolated.

### Lentiviral overexpression

Mouse wild-type and mutant Jhdm1a expression plasmids were generated by standard procedure and were fully sequenced. They were then transferred to pENTR-1A vector and recombined with pLenti-CMV/neo to generate lentiviral constructs essentially as described [43]. The titers of packaged lentiviruses were determined in liver cells. Cells were infected with similar number of viral particles, selected with G418, and cultured as above.

### Gene expression

Total RNA was extracted with Trizol reagent. Gene expression was measured by quantitative RT-PCR and normalized to internal control genes (β-actin for cells, U36b4 or cyclophilin for liver tissue). Primer sequences are provided in Table S1.

### Glucose production

Rat hepatic FAO cells expressing lentiviral Jhdm1a constructs were washed 3 times with PBS and then incubated in glucose free DMEM medium containing 2 mM sodium pyruvate and 20 mM sodium lactate for 6 hr. Glucose levels in the medium were measured with a Amplex red glucose assay kit (Invitrogen, #A22189).

### Primary mouse hepatocytes

Cells were prepared and cultured as described [24]. Cells were infected with adenoviruses at a multiplicity of infection of 50. Two days after infection, cells were starved for 6 hr in DMEM supplemented with 0.2% BSA and 2 mM sodium pyruvate before RNA isolation.

### Adenoviral infusion and animal studies

Adenoviral Jhdm1a expression and knockdown constructs and their respective control constructs were generated, and adenoviruses were produced and purified as described [42], [44]. Viral titers were determined in HEK293 cells by scoring GFP positive cells. Male wild-type C57BL/6J and *ob/ob* (on C57BL/6J background) mice were obtained from The Jackson Laboratory. Adenoviruses (4×10^9^ and 9×10^9^ viral particles for expression and knockdown, respectively) suspended in 0.2 ml PBS were injected through tail vein when animals were 10-week-old. Blood glucose levels were measured at indicated time and animals were sacrificed at Day 5. For pyruvate tolerance test, mice were fasted for 16 hr and sodium pyruvate dissolved in PBS was i.p. injected (2 g/kg body weight).

To determine the levels of liver PEPCK, G6Pase and C/EBPα protein, 50 mg liver sample were homogenized in 1 ml lysis buffer [100 mM NaCl, 50 mM Tris (pH 7.5), 0.5% Triton X-100, 5% (w/v) glycerol]. 26 µg protein extracts were separated by SDS-PAGE and probed with antibody against C/EBPα (Santa Cruz, sc-61), PEPCK (ABcam, ab28455) or G6Pase (Santa Cruz, sc-25840).

### Co-immunoprecipitation

HA-Jhdm1a and Flag-USF1 plasmids were co-transfected into Hela cells. Cells were lysed in buffer [100 mM NaCl, 50 mM Tris (pH 7.5), 0.5% Triton X-100, 5% (w/v) glycerol]. Cell extracts were incubated with anti-HA beads (Santa Cruz, sc-7392AC) for overnight and the beads were washed 4 times with buffer [100 mM NaCl, 50 mM Tris (pH 7.5), 0.1% Triton X-100, 5% glycerol]. Immunoprecipitates were probed with an anti-Flag antibody (Sigma, F7425).

### ChIP assays

Assays were performed as described [42] using antibodies against HA (Sigma, #H6908), C/EBPα (Santa Cruz, sc-61), dimethyl-H3K36 (Millipore, #07274), trimethyl-H3K36 (Abcam, #9050). Immunoprecipitate signal was normalized with input signal; both were measured by real-time QPCR. Primer sequences are provided in the Table S1.

For ChIP assays performed with liver samples, samples were generated as described with minor modifications [45]. Briefly, parts of liver from same locations were excised, cut into small pieces with a razor blade, cross-linked with 1% formaldehyde for 15 minutes at room temperature. The samples were then ground and filtered through a 40 µm cell strainer to produce a single liver cell suspension. Nuclear extracts were prepared, chromatin was sonicated using a ultrasonic processor, and immunoprecipitation was performed as described [42]. An equivalent of 40 mg of liver tissue was used for each immunoprecipitaiton. After normalized with inputs, ChIP signals were calculated as folds relative to background signal (IgG) generated from the same animal.

### Statistical analysis

Student's t test (two-tailed) was used for statistical analysis. P<0.05 was considered significant. Data are presented as mean ± s.e.m.

## Supporting Information

Figure S1 Induction of gluconeogenic gene expression by Jhdm1a knockdown. Lentiviruses expressing a second human Jhdm1a shRNA construct were infected into HepG2 cells. Cells were re-plated and selected with puromycin. Gene expression was analyzed with qRT-PCR. Data were shown as mean ± s.e.m. Targeting sequence of the second human shRNA-Jhdm1a is available on Table S1.
(PPT)

Figure S2 Gene expression by Jhdm1a knockdown in HepA1-6 cells. Lentiviruses expressing mouse Jhdm1a shRNA construct were infected into HepA1-6 cells. Cells were re-plated and selected with puromycin. Gene expression was analyzed with qRT-PCR. Data were shown as mean ± s.e.m.
(PPT)

Figure S3 Jhdm1a suppresses hormone-stimulated gluconeogenic gene expression. HepG2 cells in 12-well plates were infected with same number of lentivirus particles expressing wild type or mutant Jhdm1a. Cells were selected with G418 and treated with dibutyryl cyclic-AMP (cAMP, 0.5 mM) and dexamethasone (Dex, 1 µM) for 6 hr. Gene expression were analyzed with qRT-PCR.
(PPT)

Figure S4 Jhdm1b does not suppress gluconeogenic gene expression. HepG2 cells were infected with same number of lentivirus particles expressing Jhdm1b or vector control. Cells were selected with G418 and treated with dibutyryl cyclic-AMP (cAMP, 0.5 mM) and dexamethasone (Dex, 1 µM) for 6 hr. Gene expression were analyzed with qRT-PCR. Data are shown as mean ± s.e.m.
(PPT)

Figure S5 Adenoviral Jhdm1a shRNA constructs knock down ectopically expressed Jhdm1a. HEK293 cells in 6-well plates were infected with mouse Jhdm1a shRNA adenoviruses at an MOI of 20. HA-tagged mouse Jhdm1a expression plasmid was transfected into the cells next day. Western blot was performed to detect HA-Jhdm1a protein level with an HA antibody.
(PPT)

Figure S6 Gluconeogenic gene expression in fasted mice. Ten-week-old wild-type male C57BL/6J mice (n = 5 per group) were transduced with Jhdmla shRNA adenoviruses. Mice were fasted for 20 hr and then immediately sacrificed at Day 5. Genes expression were analyzed in liver samples with qRT-PCR. Data are shown as mean ± s.e.m.**P<0.001.
(PPT)

Figure S7 Gluconeogenic gene expression in fasted mice with a second Jhdm1a knockdown construct. Ten-week-old wild-type male C57BL/6J mice were transduced with adenoviruses expressing a second Jhdm1a knockdown construct. Mice were fasted for 20 hr and then immediately sacrificed at Day 5. Genes expression were analyzed in liver samples with qRT-PCR. (n = 5). Data are shown as mean ± s.e.m. *P<0.05, **P<0.01.
(PPT)

Figure S8 Jhdm1a expression was not affected by hormones in HepG2 cells. HepG2 cells were treated with dibutyryl cyclic-AMP (cAMP, 0.5 mM) and dexamethasone (Dex, 1 µM) or insulin (10 nM) for 5 hr. Genes expression was analyzed with qRT-PCR. Data are shown as mean ± s.e.m.
(PPT)

Figure S9 Jhdm1a does not associate with PEPCK promoter or G6Pase promoter. HepG2 cells were infected with HA-Jhdm1a or GFP adenoviruses. CHIP assays were performed with an HA antibody.
(PPT)

Figure S10 C/EBPα gene expression in fasted mice with a second Jhdm1a knockdown construct. Ten-week-old wild-type male C57BL/6J mice (n = 5) were transduced with purified adenoviruses expressing a second Jhdm1a shRNA construct. Mice were fasted for 20 hr and then immediately sacrificed at Day 5. Genes expression and protein were analyzed in liver samples. Data are shown as mean ± s.e.m. **P<0.01.
(PPT)

Figure S11 Endogenous Jhdm1a associates with the USF1-binding sites. ChIP assays were performed with an antibody against Jhdm1a (Abcam, #ab27867) in HepG2 cells expressing lentiviral Jhdm1a shRNA or scramble control. Data are shown as fold of association relative to the scramble control.
(PPT)

Figure S12 Hormonal treatment does not affect H3K36 dimethylation on C/EBPα locus in HepG2 cells. HepG2 cells were treated with dibutyryl cyclic-AMP (cAMP, 0.5 mM) and dexamethasone (Dex, 1 µM), or insulin (10 nM) for 5 hr. ChIP assays were performed with an antibody against dimethyl-H3K36.
(PPT)

Table S1 Gene full names and sequences of primers used in this study.
(DOC)
