# Supplementary figures and images for: The Histone Demethylase Jhdm1a Regulates Hepatic Gluconeogenesis
Source: PLoS Genet. 2012 Jun 14;8(6):e1002761. doi: 10.1371/journal.pgen.1002761 (PMC3375226; doi:10.1371/journal.pgen.1002761)

## Slide 1
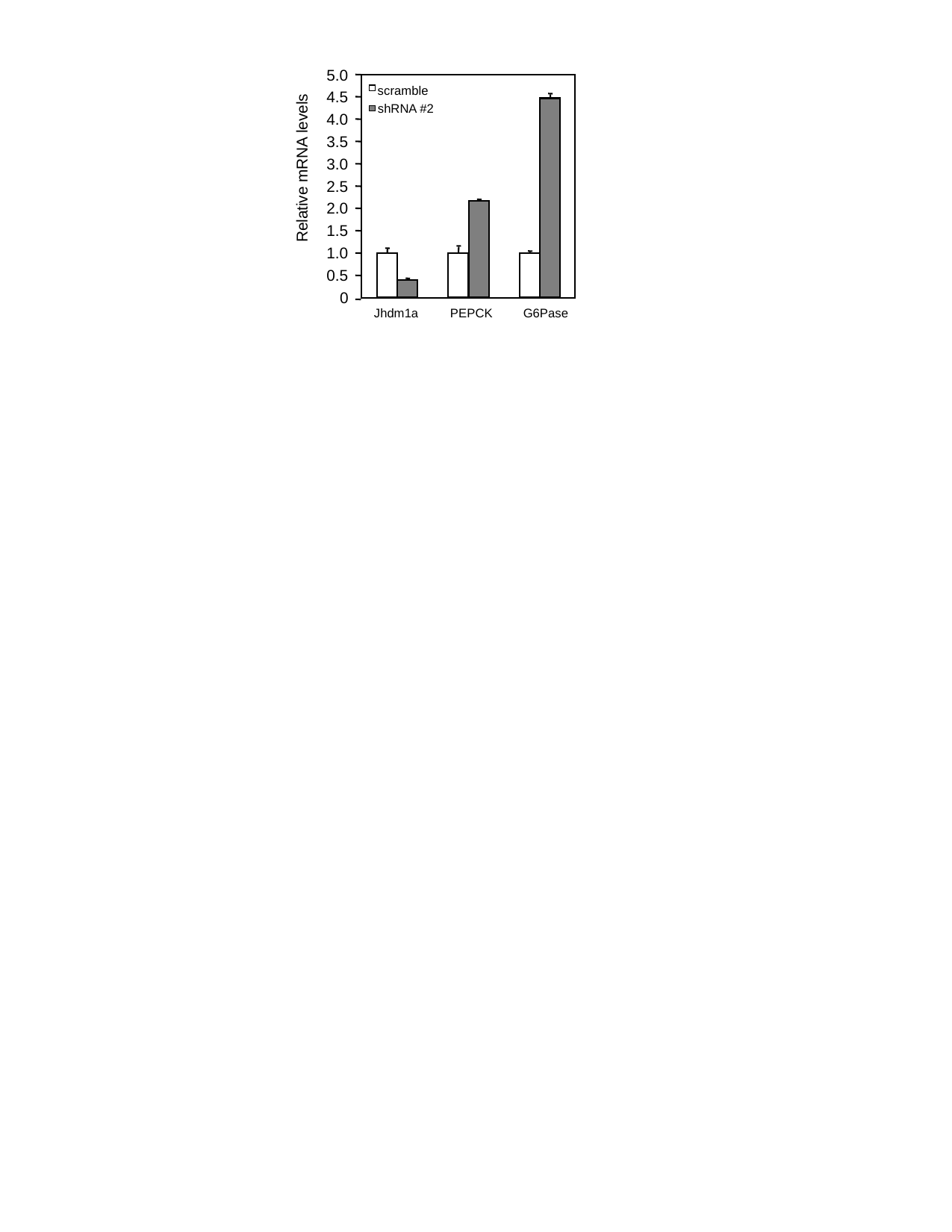

5.0
scramble
4.5
shRNA #2
4.0
3.5
Relative mRNA levels
3.0
2.5
2.0
1.5
1.0
0.5
0
Jhdm1a
PEPCK
G6Pase

Supplement: Figure S1 — Induction of gluconeogenic gene expression by Jhdm1a knockdown. Lentiviruses expressing a second human Jhdm1a shRNA construct were infected into HepG2 cells. Cells were re-plated and selected with puromycin. Gene expression was analyzed with qRT-PCR. Data were shown as mean ± s.e.m. Targeting sequence of the second human shRNA-Jhdm1a is available on Table S1. (PPT) [file pgen.1002761.s001.ppt]

## Slide 1
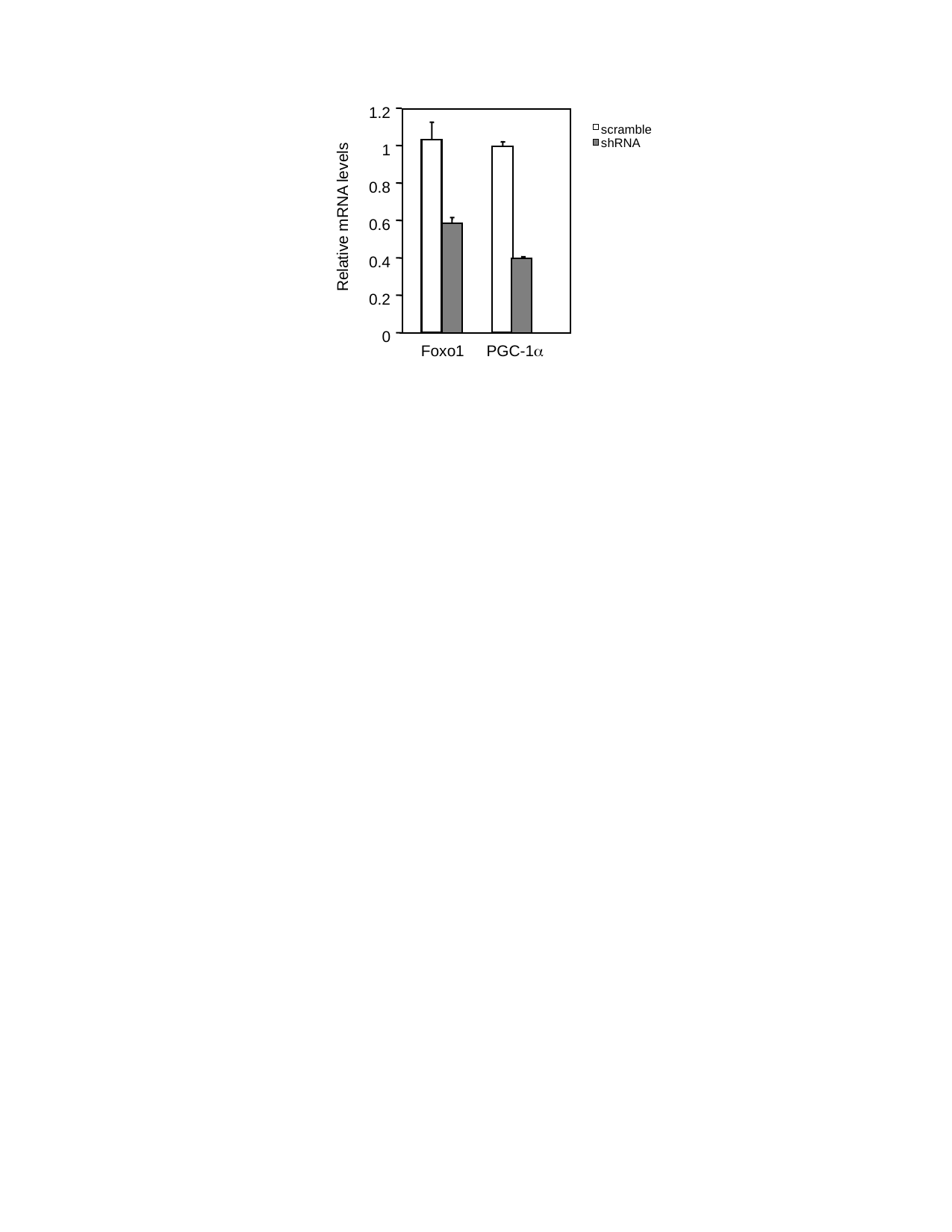

1.2
scramble
shRNA
1
0.8
Relative mRNA levels
0.6
0.4
0.2
0
Foxo1
PGC-1α

Supplement: Figure S2 — Gene expression by Jhdm1a knockdown in HepA1-6 cells. Lentiviruses expressing mouse Jhdm1a shRNA construct were infected into HepA1-6 cells. Cells were re-plated and selected with puromycin. Gene expression was analyzed with qRT-PCR. Data were shown as mean ± s.e.m. (PPT) [file pgen.1002761.s002.ppt]

## Slide 1
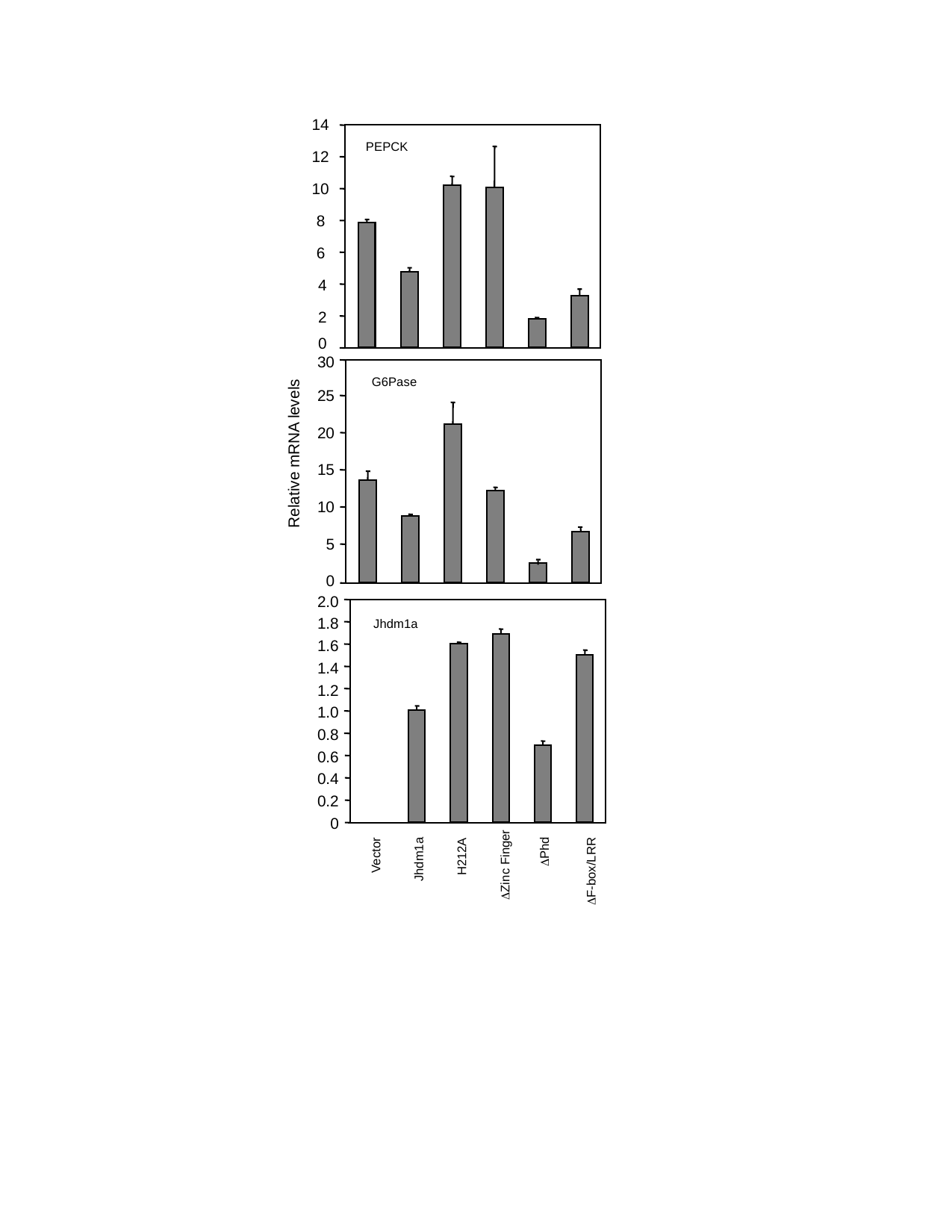

14
PEPCK
12
10
8
6
4
2
0
30
G6Pase
25
20
Relative mRNA levels
15
10
5
0
2.0
1.8
1.6
1.4
1.2
1.0
0.8
0.6
0.4
0.2
0
Jhdm1a
ΔPhd
Vector
H212A
Jhdm1a
ΔZinc Finger
ΔF-box/LRR

Supplement: Figure S3 — Jhdm1a suppresses hormone-stimulated gluconeogenic gene expression. HepG2 cells in 12-well plates were infected with same number of lentivirus particles expressing wild type or mutant Jhdm1a. Cells were selected with G418 and treated with dibutyryl cyclic-AMP (cAMP, 0.5 mM) and dexamethasone (Dex, 1 µM) for 6 hr. Gene expression were analyzed with qRT-PCR. (PPT) [file pgen.1002761.s003.ppt]

## Slide 1
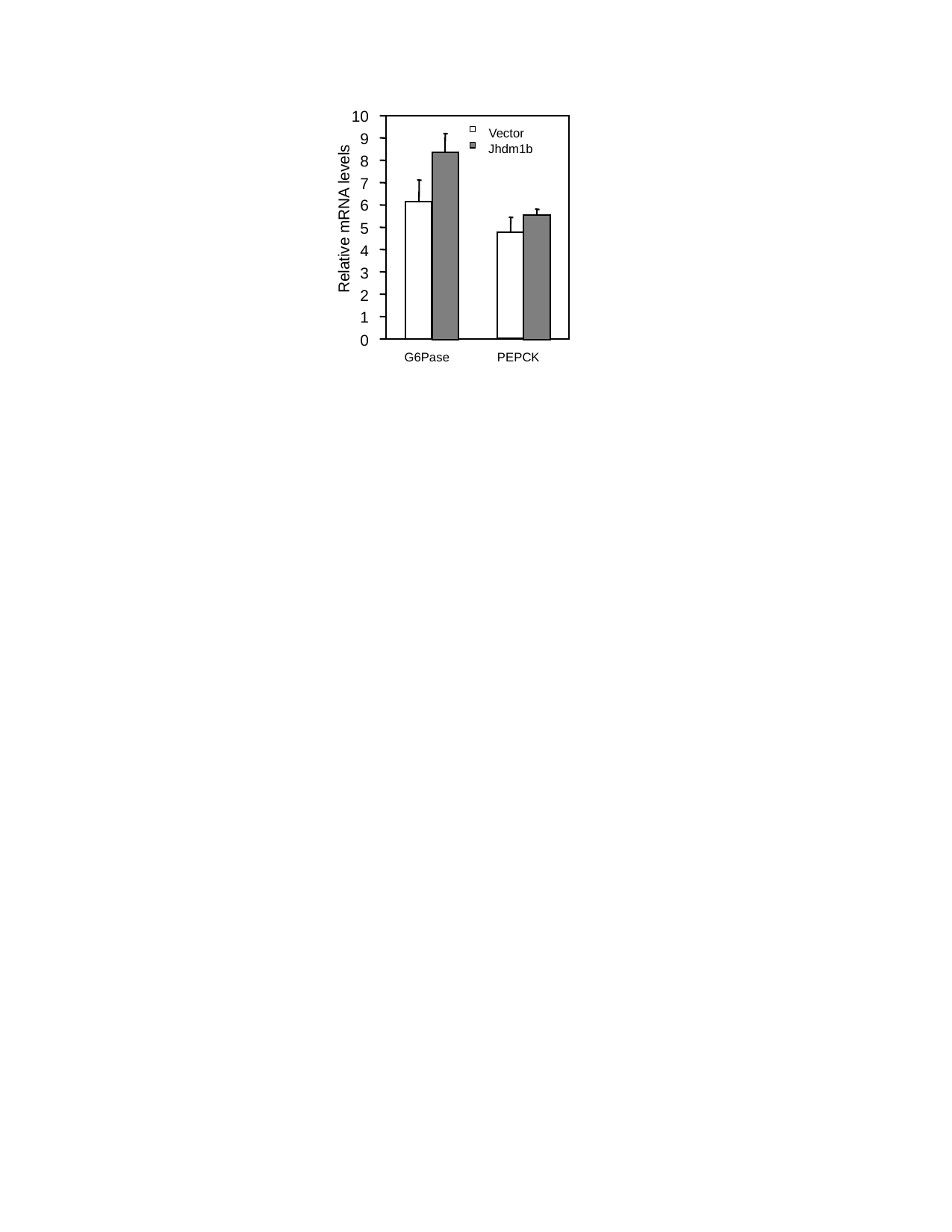

10
Vector
Jhdm1b
9
8
7
6
Relative mRNA levels
5
4
3
2
1
0
G6Pase
PEPCK

Supplement: Figure S4 — Jhdm1b does not suppress gluconeogenic gene expression. HepG2 cells were infected with same number of lentivirus particles expressing Jhdm1b or vector control. Cells were selected with G418 and treated with dibutyryl cyclic-AMP (cAMP, 0.5 mM) and dexamethasone (Dex, 1 µM) for 6 hr. Gene expression were analyzed with qRT-PCR. Data are shown as mean ± s.e.m. (PPT) [file pgen.1002761.s004.ppt]

## Slide 1
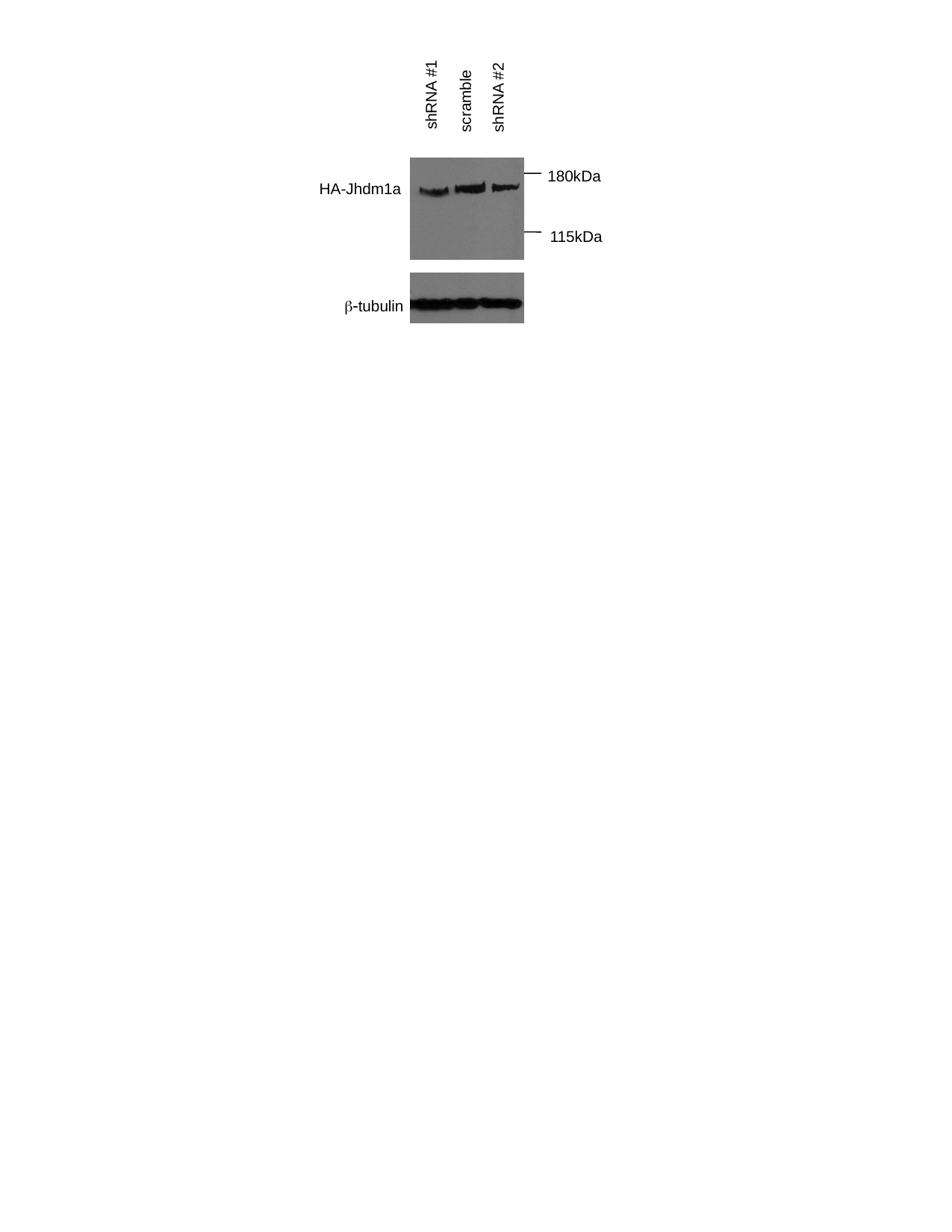

shRNA #1
shRNA #2
scramble
180kDa
HA-Jhdm1a
115kDa
β−tubulin

Supplement: Figure S5 — Adenoviral Jhdm1a shRNA constructs knock down ectopically expressed Jhdm1a. HEK293 cells in 6-well plates were infected with mouse Jhdm1a shRNA adenoviruses at an MOI of 20. HA-tagged mouse Jhdm1a expression plasmid was transfected into the cells next day. Western blot was performed to detect HA-Jhdm1a protein level with an HA antibody. (PPT) [file pgen.1002761.s005.ppt]

## Slide 1
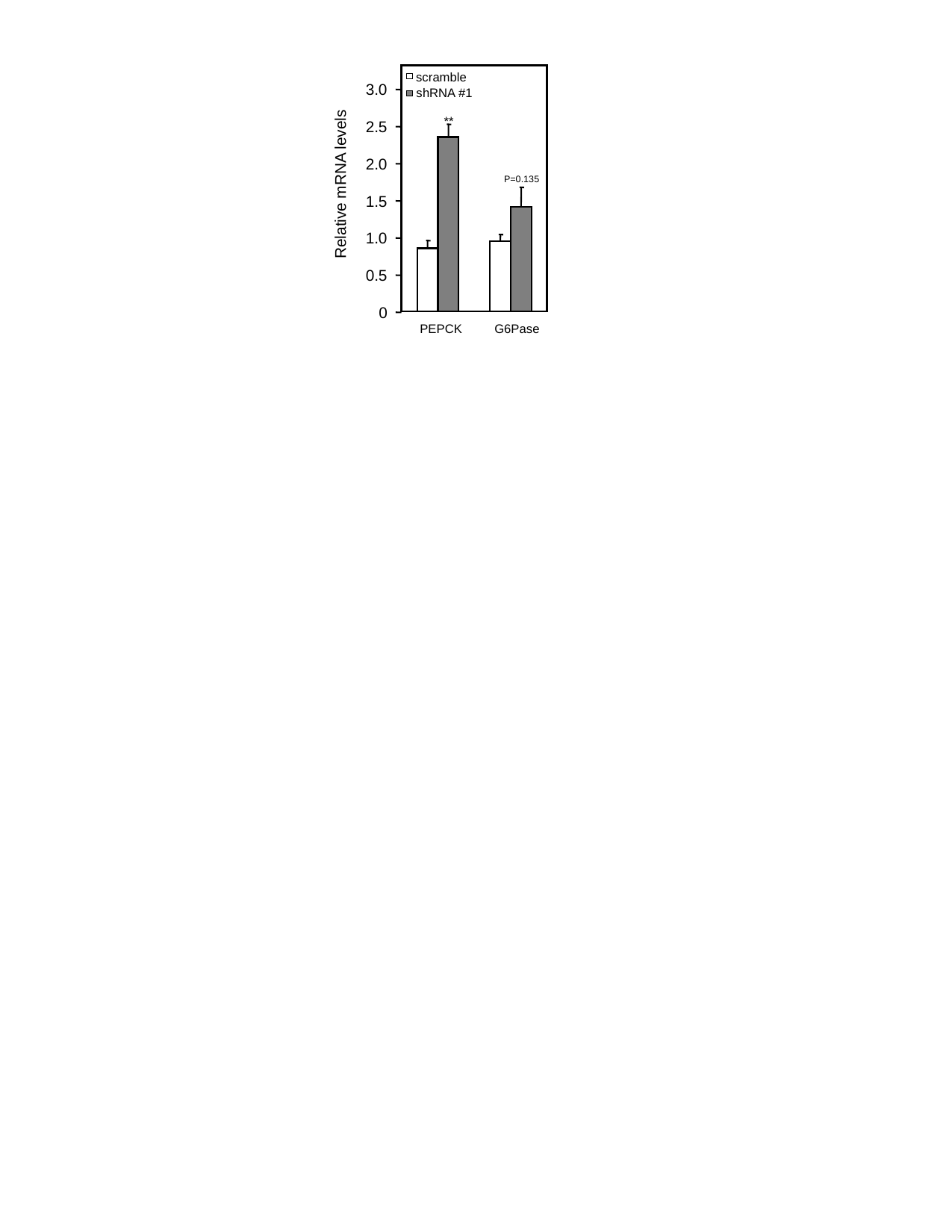

scramble
shRNA #1
3.0
**
2.5
2.0
P=0.135
1.5
1.0
0.5
0
PEPCK
G6Pase
Relative mRNA levels

Supplement: Figure S6 — Gluconeogenic gene expression in fasted mice. Ten-week-old wild-type male C57BL/6J mice (n = 5 per group) were transduced with Jhdmla shRNA adenoviruses. Mice were fasted for 20 hr and then immediately sacrificed at Day 5. Genes expression were analyzed in liver samples with qRT-PCR. Data are shown as mean ± s.e.m.**P<0.001. (PPT) [file pgen.1002761.s006.ppt]

## Slide 1
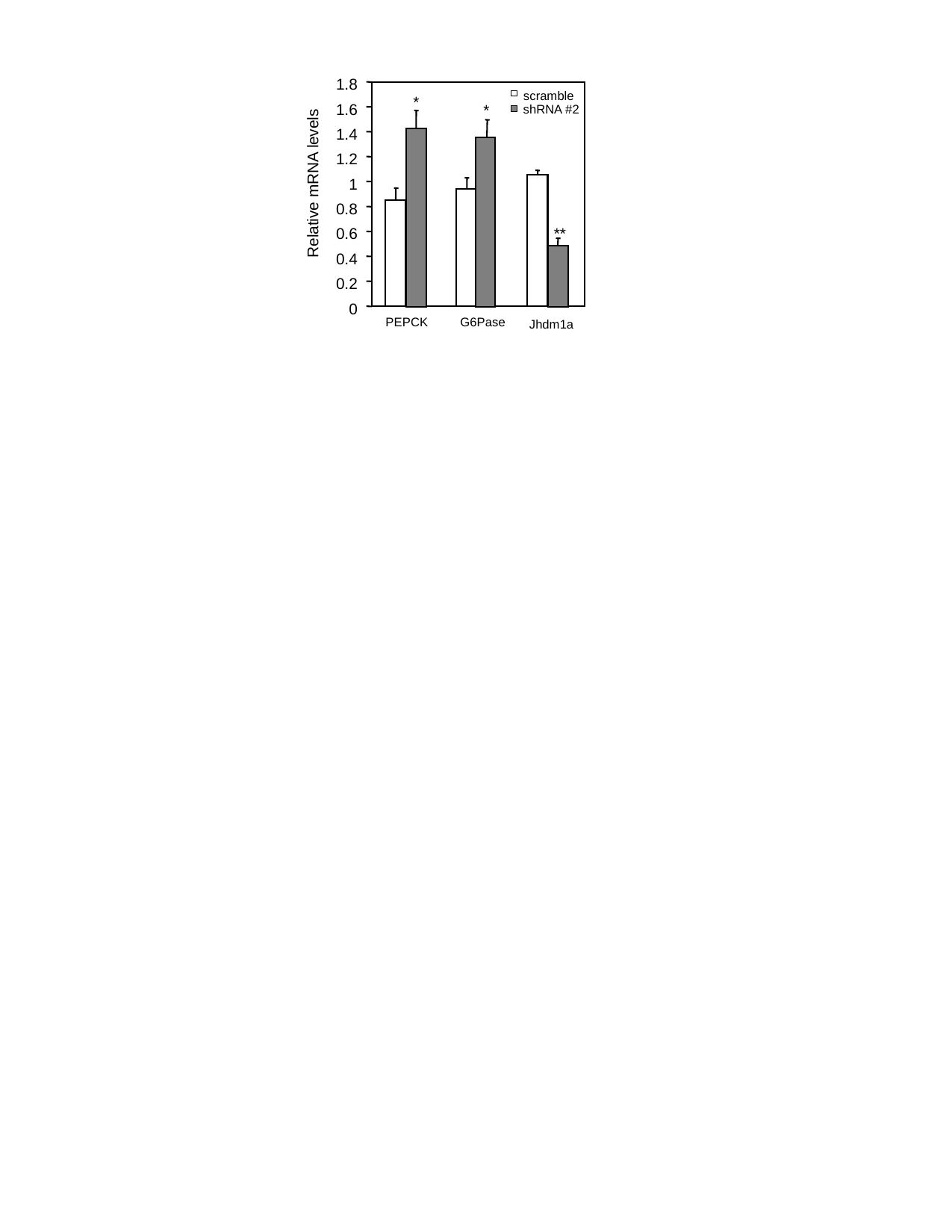

1.8
*
scramble
 shRNA #2
*
1.6
1.4
1.2
Relative mRNA levels
1
0.8
**
0.6
0.4
0.2
0
PEPCK
G6Pase
Jhdm1a

Supplement: Figure S7 — Gluconeogenic gene expression in fasted mice with a second Jhdm1a knockdown construct. Ten-week-old wild-type male C57BL/6J mice were transduced with adenoviruses expressing a second Jhdm1a knockdown construct. Mice were fasted for 20 hr and then immediately sacrificed at Day 5. Genes expression were analyzed in liver samples with qRT-PCR. (n = 5). Data are shown as mean ± s.e.m. *P<0.05, **P<0.01. (PPT) [file pgen.1002761.s007.ppt]

## Slide 1
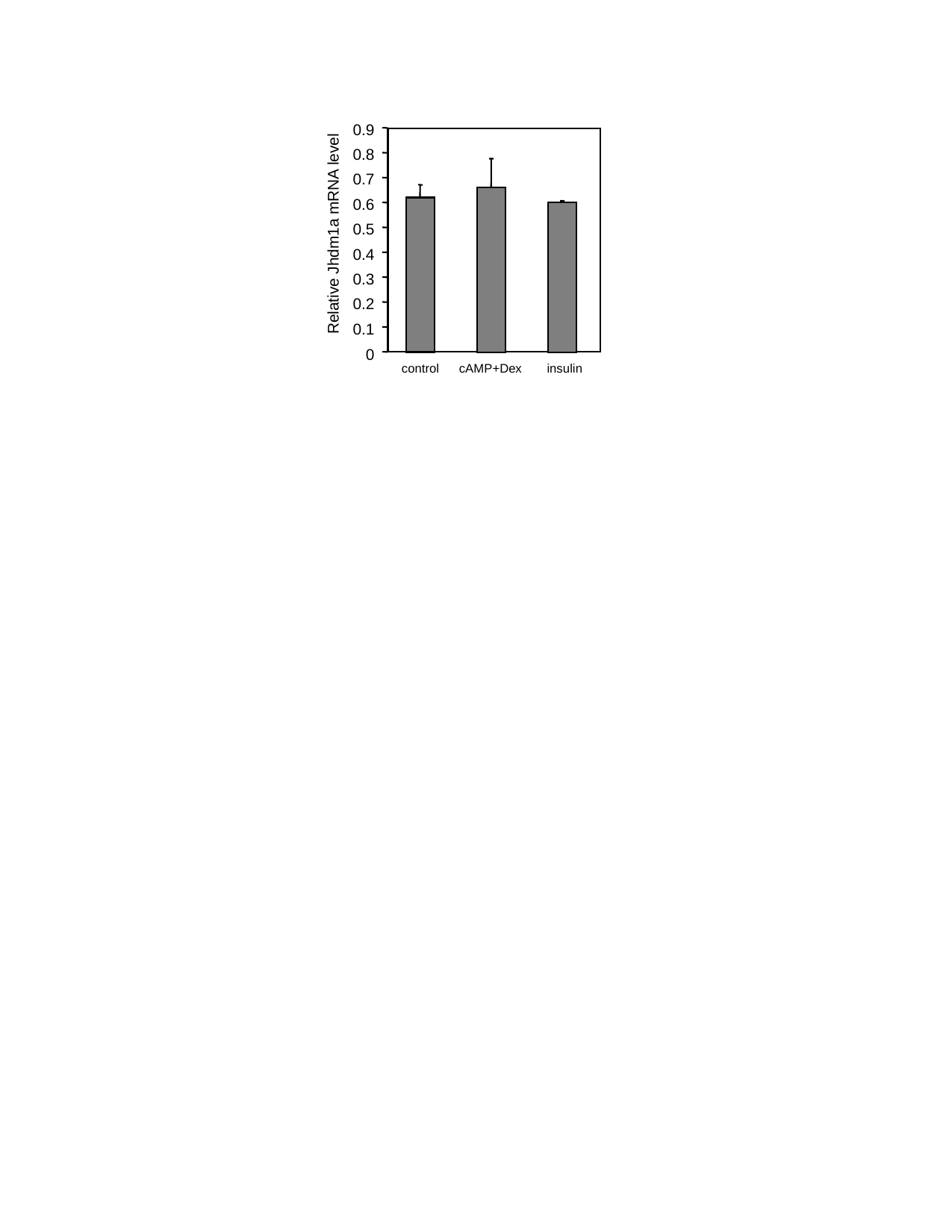

0.9
0.8
0.7
0.6
Relative Jhdm1a mRNA level
0.5
0.4
0.3
0.2
0.1
0
control
cAMP+Dex
insulin

Supplement: Figure S8 — Jhdm1a expression was not affected by hormones in HepG2 cells. HepG2 cells were treated with dibutyryl cyclic-AMP (cAMP, 0.5 mM) and dexamethasone (Dex, 1 µM) or insulin (10 nM) for 5 hr. Genes expression was analyzed with qRT-PCR. Data are shown as mean ± s.e.m. (PPT) [file pgen.1002761.s008.ppt]

## Slide 1
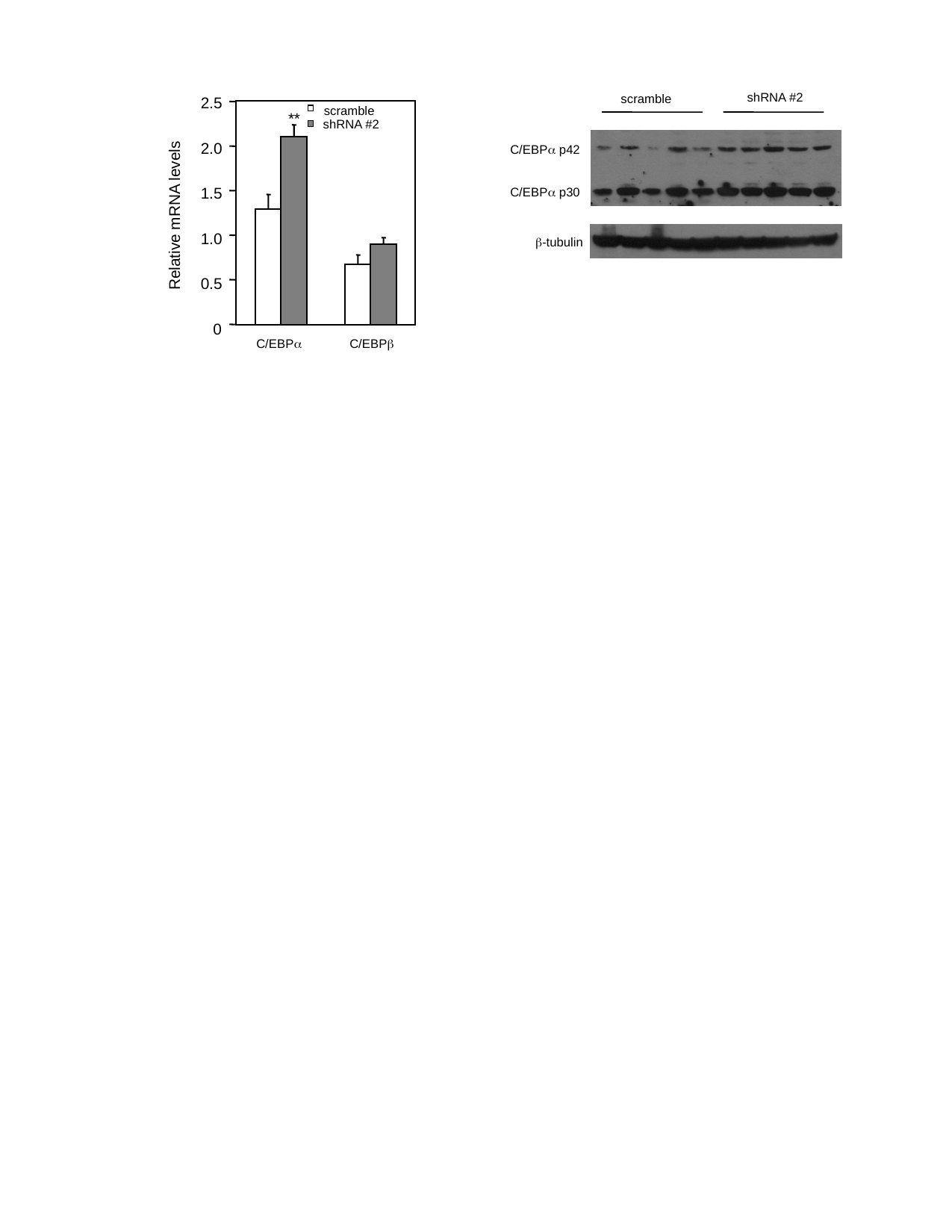

shRNA #2
scramble
2.5
2.0
1.5
1.0
0.5
0
C/EBPα
C/EBPβ
**
scramble
shRNA #2
C/EBPα p42
C/EBPα p30
Relative mRNA levels
β-tubulin

Supplement: Figure S10 — C/EBPα gene expression in fasted mice with a second Jhdm1a knockdown construct. Ten-week-old wild-type male C57BL/6J mice (n = 5) were transduced with purified adenoviruses expressing a second Jhdm1a shRNA construct. Mice were fasted for 20 hr and then immediately sacrificed at Day 5. Genes expression and protein were analyzed in liver samples. Data are shown as mean ± s.e.m. **P<0.01. (PPT) [file pgen.1002761.s010.ppt]
