# Supplementary material for: The Histone Demethylase Jhdm1a Regulates Hepatic Gluconeogenesis
Source: PLoS Genet. 2012 Jun 14;8(6):e1002761. doi: 10.1371/journal.pgen.1002761 (PMC3375226; doi:10.1371/journal.pgen.1002761)
Supplement: Figure S9 — Jhdm1a does not associate with PEPCK promoter or G6Pase promoter. HepG2 cells were infected with HA-Jhdm1a or GFP adenoviruses. CHIP assays were performed with an HA antibody. (PPT) [file pgen.1002761.s009.ppt]

## Slide 1
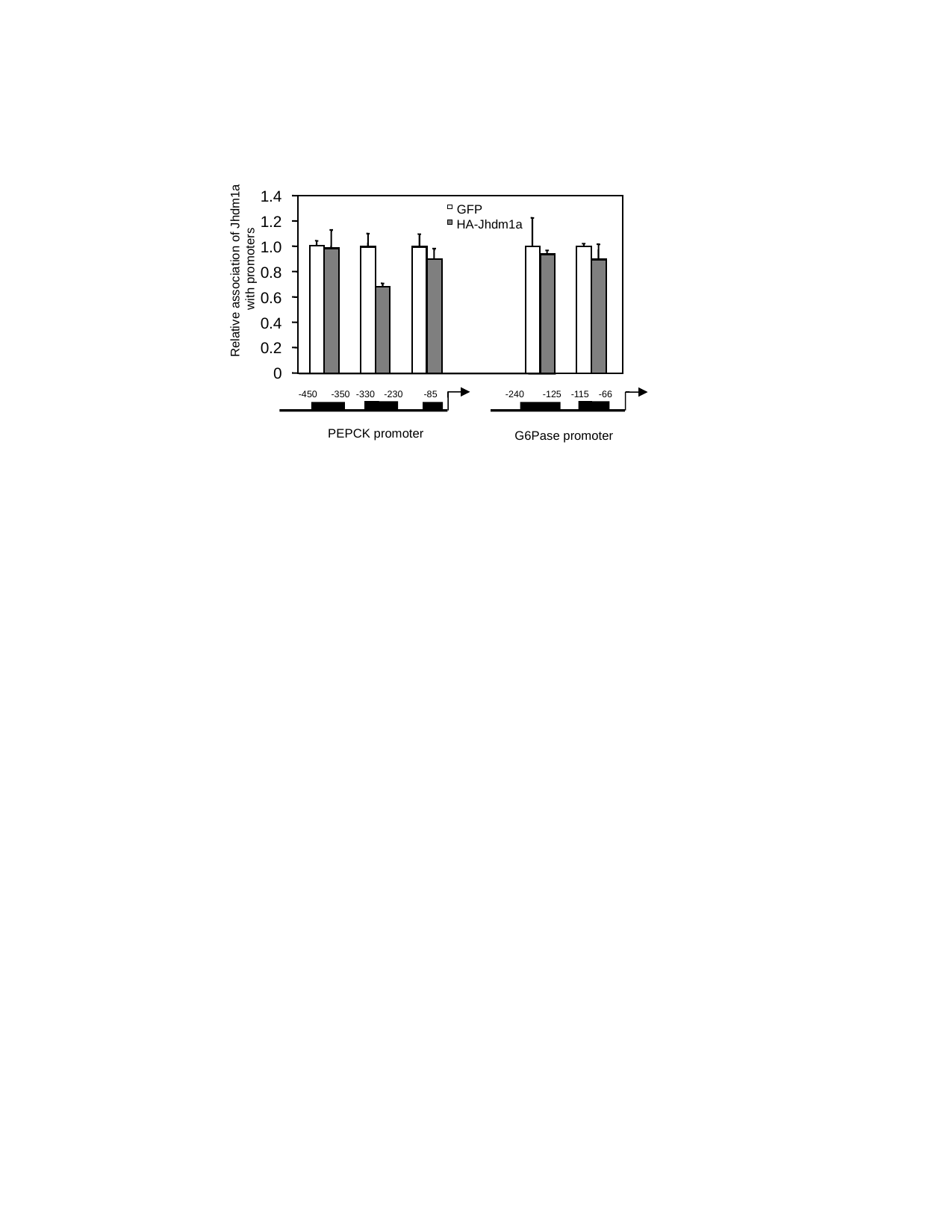

1.4
1.2
1.0
0.8
0.6
0.4
0.2
0
GFP
HA-Jhdm1a
Relative association of Jhdm1a
 with promoters
-450
-350
-330
-230
-85
-240
-125
-115
-66
PEPCK promoter
G6Pase promoter
