# Supplementary material for: The Histone Demethylase Jhdm1a Regulates Hepatic Gluconeogenesis
Source: PLoS Genet. 2012 Jun 14;8(6):e1002761. doi: 10.1371/journal.pgen.1002761 (PMC3375226; doi:10.1371/journal.pgen.1002761)
Supplement: Figure S11 — Endogenous Jhdm1a associates with the USF1-binding sites. ChIP assays were performed with an antibody against Jhdm1a (Abcam, #ab27867) in HepG2 cells expressing lentiviral Jhdm1a shRNA or scramble control. Data are shown as fold of association relative to the scramble control. (PPT) [file pgen.1002761.s011.ppt]

## Slide 1
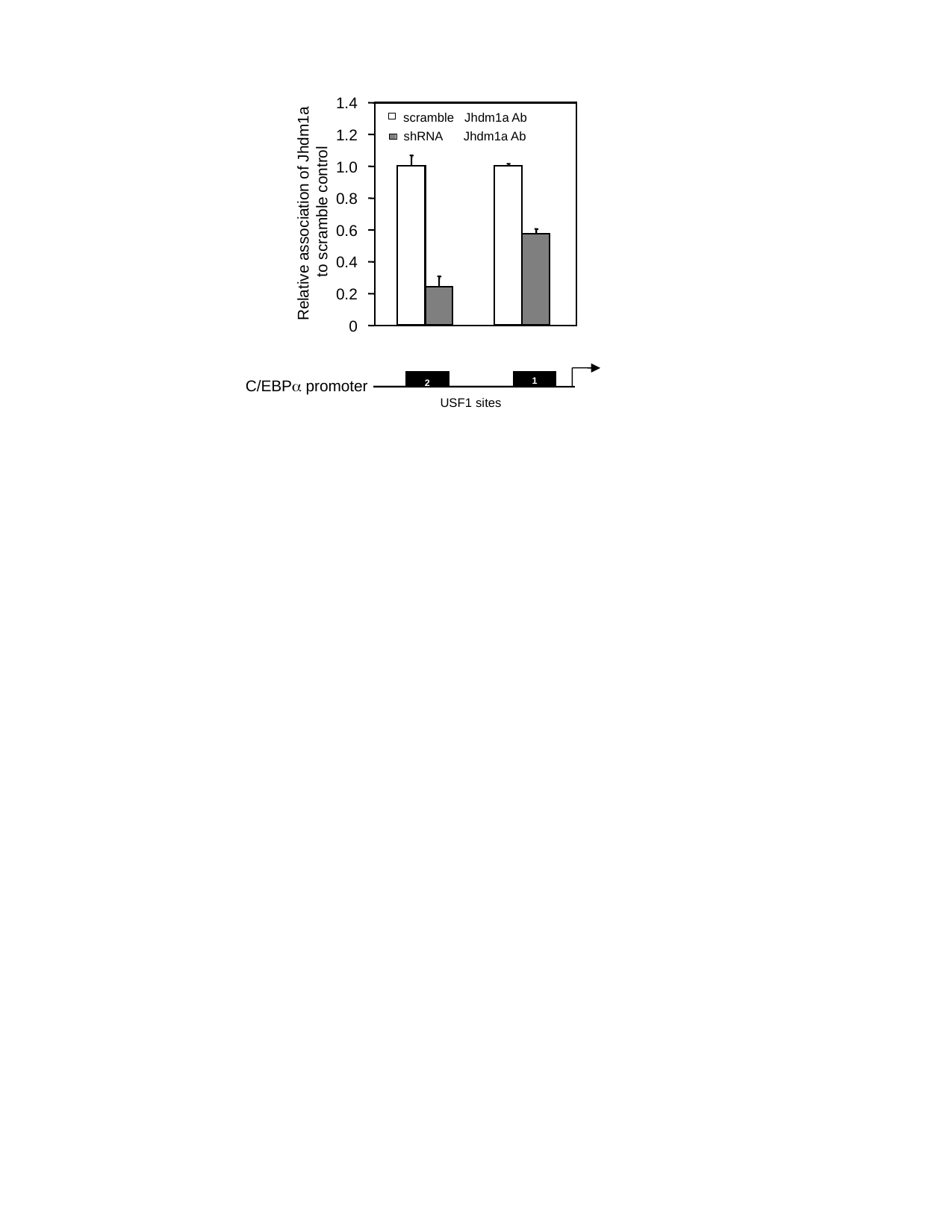

1.4
scramble Jhdm1a Ab
shRNA Jhdm1a Ab
1.2
1.0
0.8
Relative association of Jhdm1a
 to scramble control
0.6
0.4
0.2
0
1
C/EBPα promoter
2
USF1 sites
