# Supplementary material for: The Histone Demethylase Jhdm1a Regulates Hepatic Gluconeogenesis
Source: PLoS Genet. 2012 Jun 14;8(6):e1002761. doi: 10.1371/journal.pgen.1002761 (PMC3375226; doi:10.1371/journal.pgen.1002761)
Supplement: Figure S12 — Hormonal treatment does not affect H3K36 dimethylation on C/EBPα locus in HepG2 cells. HepG2 cells were treated with dibutyryl cyclic-AMP (cAMP, 0.5 mM) and dexamethasone (Dex, 1 µM), or insulin (10 nM) for 5 hr. ChIP assays were performed with an antibody against dimethyl-H3K36. (PPT) [file pgen.1002761.s012.ppt]

## Slide 1
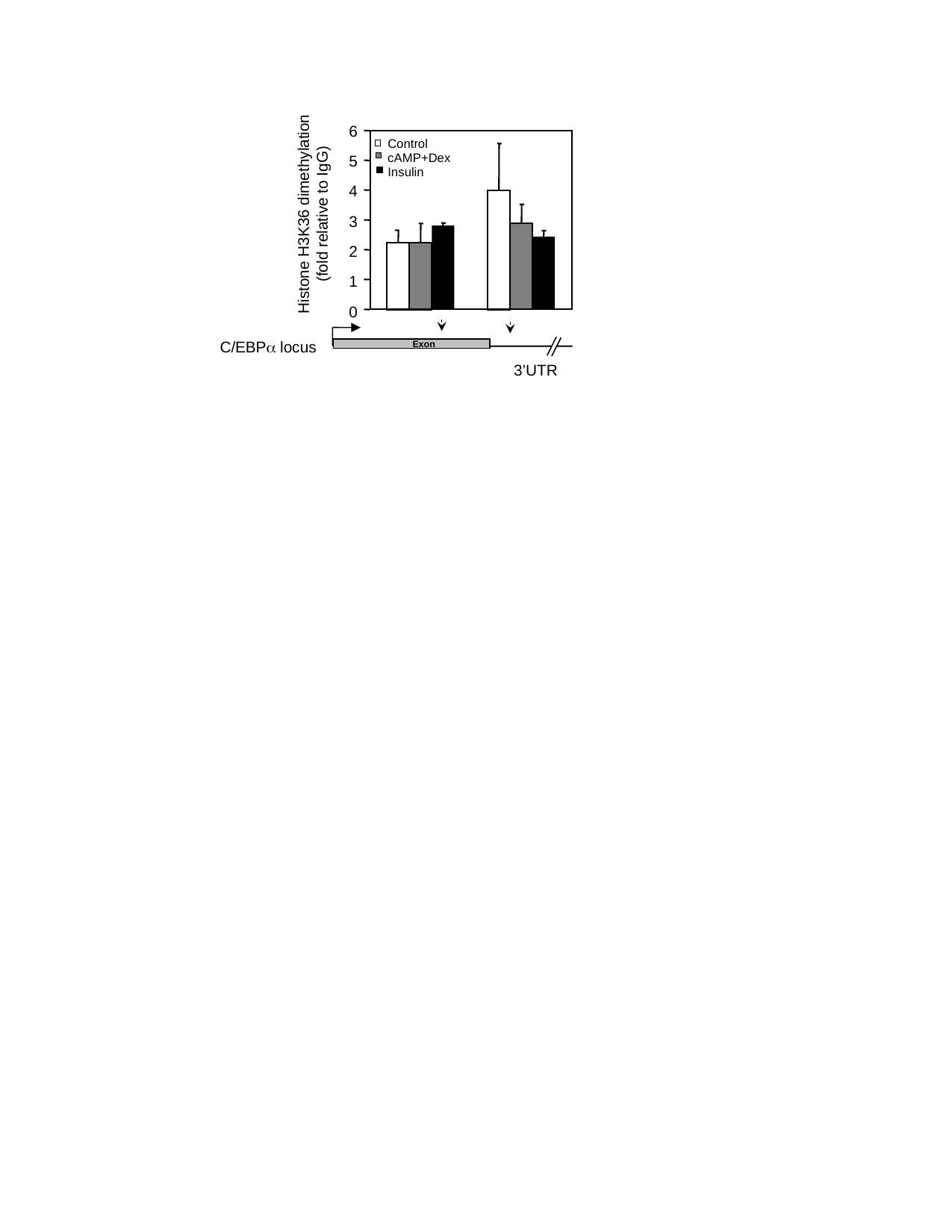

6
Control
cAMP+Dex
Insulin
5
4
Histone H3K36 dimethylation
(fold relative to IgG)
3
2
1
0
Exon
3’UTR
C/EBPα locus
