# Supplementary material for: The Histone Demethylase Jhdm1a Regulates Hepatic Gluconeogenesis
Source: PLoS Genet. 2012 Jun 14;8(6):e1002761. doi: 10.1371/journal.pgen.1002761 (PMC3375226; doi:10.1371/journal.pgen.1002761)
Supplement: Table S1 — Gene full names and sequences of primers used in this study. (DOC) [file pgen.1002761.s013.doc]

**Table S1 Gene full names and sequences of primers used in this study.**

|  | Gene full name | Sequences for shRNA, QPCR and CHIP assays |
| --- | --- | --- |
|  |  | shRNA target sequence |
| Jhdm1a | jumonji C domain-containing histone demethylase 1A | GCAGAGAACTTTGTGAATGTA (human, construct 1)  GCCCTCACTGGAGTTCCTATA (human, construct 2)  CCCACAGGAATAGAAGACGAA (mouse, construct 1)  GCTCCAAACCAACAAATATAA (mouse, construct 2) |
| C/EBPα | CCAAT/enhancer binding protein, alpha | GCTGGAGCTGACCAGTGACAA |
| USF1 | upstream transcription factor 1 | CCACGGATTAGAGGTCGTCAT |
|  |  | Primers used for real-time QPCR |
| Jhdm1a | jumonji C domain-containing histone demethylase 1A | F: GATGTCTGTCTTCCGCTACCTC (human)  R: GCACAATGGCCTTACACCTACT (human)  F: ACACCAGGCTTGAGAATATGGT (mouse)  R: GGCATTTGTTGATTCTGTCTGA (mouse) |
| PEPCK | phosphoenolpyruvate carboxykinase 1 (soluble) | F: TGACAACTGCTGGTTGGCT (human)  R: TGGTGCGACCTTTCATGC (human)  F: TCTCTGATCCAGACCTTCCAA (mouse)  R: GAAGTCCAGACCGTTATGCAG (mouse) |
| G6Pase | glucose-6-phosphatase, catalytic subunit | F: GGGAAAGATAAAGCCGACCTAC (human)  R: CAGCAAGGTAGATTCGTGACAG (human)  F: AAGCCAACGTATGGATTCCG (mouse)  R: ACAGCAATGCCTGACAAGACT (mouse) |
| FBP1 | fructose-1,6-bisphosphatase 1 | F: CCCCAGATAATTCAGCTCCTTA  R: GTTGCATTCGTACAGCAGTCTC |
| FAS | fatty acid synthase | F: AGTTCACGGACATGGAGCACAACA  R: ATGGTACTTGGCCTTGGGTGTGTA |
| SCD1 | stearoyl-CoA desaturase (delta-9-desaturase) | F: CCACCGCTCTTACAAAGCTC  R: CACGAGCCCATTCATAGACA |
| ACC1 | acetyl-CoA carboxylase alpha | F: AGCTGGAGCCCTCAACAAA  R: GGACAGTGAAAACCCCTTCA |
| LDLR | low density lipoprotein receptor | F: AGGCTGTGGGCTCCATAGG  R: TGCGGTCCAGGGTCATCT |
| MCAD | acyl-CoA dehydrogenase, C-4 to C-12 straight chain | F: CCAAGTATGCCCTGGAAAGGAAAA  R: CATTGCCATTTCAGCCAGCATAAA |
| ATP5A1 | ATP synthase, H+ transporting, mitochondrial F1 complex, alpha subunit 1, cardiac muscle | F: ATGGGCTGAGGAATGTTCAA  R: CCAAACACGACAACACCAAC |
| ATP5B | ATP synthase, H+ transporting, mitochondrial F1 complex, beta polypeptide | F: CATGGGTACTATGCAGGAAAGA  R: GGCAGGGTCAGTCAAGTCAT |
| UCP2 | uncoupling protein 2 (mitochondrial, proton carrier) | F: GAGATACCAAAGCACCGTCAAT  R: TGATGAGGTCATAGGTCACCAG |
| PFKL | phosphofructokinase, liver | F: GGCTGGGAGAACTTCATGTG  R: TCACGTAGCTGGACGAGATG |
| PYGL | phosphorylase, glycogen, liver | F: GAGACTTGCTGCCTGCTTCT  R: GCCATCCATCTCGGATCTT |
| AGL | amylo-alpha-1, 6-glucosidase, 4-alpha-glucanotransferase | F: GGATGGGTAATGGGAGATGA  R: TAACACTGTCTCCCCAGCAA |
| PGM1 | phosphoglucomutase 1 | F: GTATGGCCGGAATTTCTTCA  R: GAGAACTGCTTCCCCACAAA |
| HNF4α | hepatocyte nuclear factor 4, alpha | F: GTGGTGGACAAAGACAAGAGG  R: CATAGCTTGACCTTCGAGTGC |
| GR | nuclear receptor subfamily 3, group C, member 1 (glucocorticoid receptor) | F: ACTTCCCTGGTCGAACAGTTT  R: GAAGCTTCATCAGAGCACACC |
| FOXO1 | forkhead box O1 | F: TACGAGTGGATGGTCAAGAGC (human)  R: TTCTGCACACGAATGAACTTG (human)  F: AAGAGCGTGCCCTACTTCAA (mouse)  R: TGCTGTGAAGGGACAGATTG (mouse) |
| FOXA2 | forkhead box A2 | F: CCGACTGGAGCAGCTACTATG  R: TACGTGTTCATGCCGTTCAT |
| C/EBPα | CCAAT/enhancer binding protein (C/EBP), alpha | F: TGGACAAGAACAGCAACGAGTA (human)  R: ATTGTCACTGGTCAGCTCCAG (human)  F: AAACAACGCAACGTGGAGAC (mouse)  R: TGTCCAGTTCACGGCTCAG (mouse) |
| C/EBPβ | CCAAT/enhancer binding protein (C/EBP), beta | F: ACTTCAGCCCGTACCTGGAG (human)  R: GAGAAGAGGTCGGAGAGGAAGT (human)  F: GGGTTGTTGATGTTTTTGGTTT (mouse)  R: GAAACGGAAAAGGTTCTCAAAA (mouse) |
| CREB | cAMP responsive element binding protein 1 | F: TGCCAACTCCAATTTACCAA  R: ACCCCATCGGTACCATTGTT |
| NURR1 | nuclear receptor subfamily 4, group A, member 2 | F: CCATTGTTGAATTCTCCTCCA  R: TTCCACTCTCTTGGGTTCCTT |
| SIRT1 | sirtuin 1 | F: GTATTTATGCTCGCCTTGCTG  R: TGACAGAGAGATGGCTGGAAT |
| PGC-1α | peroxisome proliferator-activated receptor gamma, coactivator 1 alpha | F: CACAGTCGCAGTCACAACACT (human)  R: TTCCACACTTAAGGTGCGTTC (human)  F: AACCACACCCACAGGATCAGA (mouse)  R: TCTTCGCTTTATTGCTCCATGA (mouse) |
| TORC2 | CREB regulated transcription coactivator 2 | F: GGGCAACAGTACCTCCAATTT  R: GTGGCTGAGAGGTGAATGAAG |
| P300 | E1A binding protein p300 | F: GCCACCATGGAGAAGCATAA  R: AGATCGCAGGGGATGAGAG |
| USF1 | upstream transcription factor 1 | F: CCTGGCACTGGTCAATTCTT  R: GGGGAATAAGGGTGAGTCCT |
| β-actin | actin, beta | F: CCTGGCACCCAGCACAAT  R: GCCGATCCACACACGGAGTACT |
| Cyclophilin B | peptidylprolyl isomerase B | F: TGGAGAGCACCAAGACAGACA  R: TGCCGGAGTCGACAATGAT |
| U36B4 | Ribosomal protein, large, P0 | F: AGATGCAGCAGATCCGCA  R: GTTCTTGCCCATCAGCACC |
|  |  | CHIP assays primers |
| PEPCK | Promoter -426~-316  Promoter -340~-233  Promoter -127~-63 | F: GTGCATCCTTCCCATGAACT  R: TGCTAGCAGCACATTTTGTGT  F: TGGTACACAAAATGTGCTGCT  R: GACCCATTTTACTGCTGTTGC  F: GGGCGTGTTGCAGTGAGT  R: GGGCAGGTCTCTGGATCA |
| G6Pase | Promoter -242~-132  Promoter -150~-58 | F: CCAAGAAGCATGCCAAAGTT  R: TGCAAACATGTTCAGGGTGA  F: CACCCTGAACATGTTTGCAT  R: AGCCCTGATCTTTGGACTCA |
| C/EBPα | Promoter -1428~-1288  Promoter -1233~-1146  Promoter -1029~-916  Promoter -931~-808  Promoter -318~-269  Exon +638~+746  Exon +921~+1003  3’UTR +1281~+1381  3’UTR +2081~+2198 | F: TAAGGCCACTGTCGGTGAAG  R: GAGCCCTCAAGTGTCTCCTG  F: TAAAGAATGCGAGGGACGCA  R: TTGAGTGCGGCAGCTAATGCT  F: ACACCCTCGCTCCCGCCGTT  R: TTAACTCTGGGGCCGGGATT  F: CCGGCCCCAGAGTTAAGTT  R: GTGGAGTCGCCGATTTTT  F: CCTAGGGCAGGCAGGAGGAGG  R: CGCGCCCACGTGGTCGGTA  F: CGGCCTCTTCCCTTACCA  R: GTGCGCGATCTGGAACTG  F: CGTGGAGACGCAGCAGAA  R: GTTCGCGGCTCAGCTGTT  F: GCGAGCCAGGACTAGGAGAT  R: CCTCATCTTAGACGCACCAAGT  F: CAGAGGGACCGGAGTTATGA  R: AGACGCGCACATTCACATT |
| C/EBPβ | Exon +540~+622  Exon +1082~+1156  3’UTR +1324~+1426 | F: CGACTTCCTCTCCGACCTCT  R: GTGCCAAGCATTCTACAGCA  F: AACCTGGAGACGCAGCAC  R: CAGCTGCTCCACCTTCTTCT  F: CGGCAAAACTTTGGCACT  R: CGGTTGGTTTGGACAAAAAT |
